# Air Pollution and Cardiac Remodeling and Function in Patients With Breast Cancer

**DOI:** 10.1001/jamanetworkopen.2025.52323

**Published:** 2026-01-15

**Authors:** Wonyoung Jung, Kyunga Ko, Amanda M. Smith, Anran Huang, Congying Xia, Yehoda M. Martei, Vivek K. Narayan, Amy S. Clark, Benedicte Lefebvre, Kellie McDermott, Daniel Koropeckyj-Cox, Omotayo Fasan, Alexander Hutsell, Amber Daniels, Virginia Englefield, Kasey J. Leger, Kelly D. Getz, Hari K. Narayan, Julian D. Marshall, Tiffany M. Powell-Wiley, Clyde W. Yancy, Bonnie Ky

**Affiliations:** 1 Division of Cardiology, Department of Medicine, Perelman School of Medicine, University of Pennsylvania, Philadelphia; 2 Thalheimer Center for Cardio-Oncology, Abramson Cancer Center, Perelman School of Medicine at the University of Pennsylvania, Philadelphia; 3 Division of Hematology-Oncology, Hospital of the University of Pennsylvania, Philadelphia; 4 Department of Pediatrics, Seattle Children’s Hospital, University of Washington School of Medicine, Seattle; 5 Department of Biostatistics, Epidemiology and Informatics, Perelman School of Medicine, University of Pennsylvania, Philadelphia; 6 Department of Pediatrics, University of California San Diego, La Jolla; 7 Department of Civil & Environmental Engineering, University of Washington, Seattle; 8 Social Determinants of Obesity and Cardiovascular Risk Laboratory, Cardiovascular Branch, Division of Intramural Research, National Heart, Lung, and Blood Institute, National Institutes of Health, Bethesda, Maryland; 9 Intramural Research Program, National Institute on Minority Health and Health Disparities, National Institutes of Health, Bethesda, Maryland; 10 Department of Internal Medicine, Division of Cardiology, Northwestern University, Feinberg School of Medicine, Chicago, Illinois

## Abstract

**Importance:**

The relationship between air pollution and cardiac remodeling in patients with cancer treated with cardiotoxic therapy is undefined.

**Objective:**

To assess the associations between air pollutants and changes in cardiac function, structure, and remodeling in patients with breast cancer treated with anthracyclines and/or trastuzumab therapy.

**Design, Setting, and Participants:**

This longitudinal prospective cohort study included patients with breast cancer enrolled at multiple sites of a quaternary health care system from July 1, 2010, to November 1, 2018. All participants were initiating anthracyclines and/or trastuzumab. Data were analyzed from December 1, 2024, to April 30, 2025.

**Exposures:**

Three-year average census tract–level concentrations of fine particulate matter with diameter of 2.5 µm or less (PM_2.5_), particulate matter with diameter of 10 µm or less (PM_10_), nitrogen dioxide (NO_2_), and ozone (O_3_).

**Main Outcomes and Measures:**

Core laboratory–quantified, echocardiography-derived measures of cardiac remodeling and function and incidence of cardiac dysfunction, defined as a left ventricular ejection fraction (LVEF) decline of 10% or more from baseline to less than 50%. Multivariable linear regression and generalized estimating equations defined the cross-sectional and longitudinal associations between air pollution and measures of cardiac remodeling and function. Cause-specific hazard models defined the adjusted associations between air pollution and cardiac dysfunction.

**Results:**

Across 580 female patients (median age, 50 years [IQR, 42-58 years]), 3642 echocardiograms were obtained at standardized time intervals over a median of 3.1 years (IQR, 2.3-3.6 years) and centrally quantified. Cardiac dysfunction was observed in 98 of 574 participants (17.1%). Concentrations of PM_2.5_ (median, 9.26 μg/m^3^ [IQR, 8.49-10.17 μg/m^3^]) and O_3_ (median, 47.00 parts per billion [ppb] [IQR, 45.50-48.19 ppb]) were each associated with cardiac dysfunction and adverse remodeling, cross-sectionally and longitudinally. Over time, each IQR-increment increase in PM_2.5_ (1.68 μg/m^3^) and O_3_ (2.69 ppb) was associated with a mean LVEF change of −1.3% (95% CI, −1.8% to −0.8%) and −1.4% (95% CI, −1.8% to −1.0%), respectively; worse longitudinal strain (−1.0% [95% CI, −1.3% to −0.7%] and −1.1% [95% CI, −1.3% to −0.8%], respectively); and left ventricular mass increase of 4.8 g/m^2^ (95% CI, 3.1-6.5 g/m^2^) and 3.2 g/m^2^ (95% CI, 2.1-4.3 g/m^2^), respectively. Patients in the highest tertiles of PM_2.5_ (adjusted hazard ratio [AHR], 2.03; 95% CI, 1.17-3.52) and O_3_ (AHR, 2.15; 95% CI, 1.23-3.78) exposure were at a significantly higher risk of cardiac dysfunction compared with those in the lowest tertile. Neither PM_10_ (AHR, 0.84; 95% CI, 0.49-1.44) nor NO_2_ (AHR, 0.92; 95% CI, 0.50-1.70) showed significant associations with cardiac dysfunction.

**Conclusions and Relevance:**

In this cohort study, PM_2.5_ and O_3_ exposure was independently associated with worse cardiac remodeling and function in patients with breast cancer treated with cardiotoxic therapy. These findings highlight the importance of modifying environmental exposures to mitigate cardiovascular disease risk.

## Key Points

**Question:**

Is exposure to air pollutants associated with cardiac remodeling and function among women with breast cancer treated with potentially cardiotoxic therapies?

**Findings:**

In a cohort study of 580 patients with breast cancer treated with anthracyclines and/or trastuzumab, greater exposure to fine particulate matter with diameter of 2.5 µm or less and ozone was associated with worse cardiac remodeling and function and greater risk of therapy-related cardiac dysfunction. Exposure to particulate matter with diameter of 10 µm or less and nitrogen dioxide showed limited associations.

**Meaning:**

This study’s comprehensive structural and functional data support the critical need for strategies to reduce fine particulate matter and ozone exposure, particularly in patients with breast cancer.

## Teaser

This cohort study assesses associations between exposure to air pollutants and changes in cardiac structure, function, and remodeling in patients with breast cancer treated with potentially cardiotoxic therapies.

## Introduction

Cardiac dysfunction remains an important concern among patients with breast cancer treated with anthracyclines and/or trastuzumab. Although these therapies have substantially improved cancer survival,^1^ they are also associated with a risk of adverse cardiac remodeling, declines in left ventricular (LV) ejection fraction (LVEF), and clinically overt heart failure (HF).^2,3,4,5^ Identification and mitigation of potentially modifiable cardiovascular risk factors is thus important for improving the cardiovascular health of this growing population. While much of the field of cardio-oncology has focused on clinical cardiovascular risk factors (eg, hypertension, diabetes, and hyperlipidemia), the associations between environmental factors and cardiac function and remodeling in cancer remain undefined.

Air pollution has emerged as a major contributor to global morbidity and mortality, with nearly 6.7 million deaths worldwide attributed to air pollution in 2019 alone.^6^ In the general population, pollutants such as fine particulate matter with diameter of 2.5 µm or less (PM_2.5_), particulate matter with diameter of 10 µm or less (PM_10_), and gaseous emissions (nitrogen dioxide [NO_2_], ozone [O_3_]) are factors associated with worse cardiovascular outcomes and adverse cardiac remodeling and diastolic dysfunction, with the most robust epidemiologic evidence observed with long-term exposure to PM_2.5_.^7^ Each 10 μg/m^3^ increase in PM_2.5_ was associated with a 74.8% higher risk of HF.^8^ In a large, population-based US cohort study of patients with cancer and cancer survivors, PM_2.5_ was associated with 1.32-fold higher cardiovascular mortality, which was worsened in patients who received chemotherapy or radiation.^9^ However, understanding of air pollution and the relationships with detailed measures of cardiac remodeling and function, particularly in patients with breast cancer treated with potentially cardiotoxic cancer therapy, remains limited.

The objective of this study was thus to determine the associations between air pollutant exposure and changes in cardiac function, structure, and remodeling, defined by comprehensive quantitative echocardiography in patients with breast cancer treated with anthracyclines and/or trastuzumab therapy. Given previously established associations in the individual domains of cancer and cardiovascular disease (CVD),^10^ we hypothesized that PM_2.5_ levels would be most strongly associated with cardiac dysfunction and adverse remodeling.

## Methods

### Study Cohort

This longitudinal prospective cohort study used data from the Cardiotoxicity of Cancer Therapy randomized clinical trial, which enrolled women (aged ≥18 years) diagnosed with breast cancer between July 1, 2010, and November 1, 2018, from the Abramson Cancer Center at the University of Pennsylvania, a quaternary health care system, as previously described.^11,12,13,14,15^ The study was approved by the University of Pennsylvania’s institutional review board and adhered to the principles of the Declaration of Helsinki.^16^ All participants provided written informed consent. The study adhered to the Strengthening the Reporting of Observational Studies in Epidemiology (STROBE) reporting guideline.

The primary inclusion criterion was planned receipt of adjuvant or neoadjuvant cancer therapy with anthracyclines and/or trastuzumab. The exclusion criteria included pregnancy or an inability or unwillingness to provide informed consent. The cancer therapy regimen was determined by the treating oncologist and consisted of doxorubicin (240 mg/m^2^ divided into 4 cycles of 60 mg/m^2^ each) and cyclophosphamide followed by paclitaxel (hereafter, *doxorubicin*), doxorubicin (240 mg/m^2^ divided into 4 cycles) and cyclophosphamide followed by paclitaxel and trastuzumab (hereafter, *doxorubicin plus trastuzumab*), or trastuzumab with docetaxel and carboplatin (hereafter, *trastuzumab*).

Study visits occurred prior to cancer therapy initiation (baseline) and at standardized time intervals thereafter (eFigure 1 in Supplement 1). At each study visit, a questionnaire related to medical history was administered and echocardiography was performed according to a standardized imaging protocol. Clinical covariate data were obtained from participant interview and by review of the electronic medical records. Race, self-reported by each study participant, was obtained secondary to National Institutes of Health reporting guidelines. The Social Vulnerability Index (SVI) was determined using each participant’s baseline residential address, which was linked to the corresponding census tract identifier and matched to the SVI data developed by the Centers for Disease Control and Prevention (CDC) and Agency for Toxic Substances and Disease Registry.^17^

### Exposure Assessment

We evaluated census tract–level exposure to PM_2.5_, PM_10_, NO_2_, and O_3_ based on 3-year average pollutant concentrations prior to cancer therapy initiation. For PM_2.5_, PM_10_, and NO_2_ we used annual averages, while for O_3_ we used the average of the daily maximum 8-hour moving value from May through September (warm-season ozone concentration).^18^ All estimates were derived from a land-use regression model developed by the Center for Air, Climate, and Energy Solutions, which integrates data from Federal Reference Method monitors, and Integrated Monitoring of Protected Visual Environments sites.^18^ In the model, approximately 350 geographic covariates (eg, traffic densities, land use, and satellite-based indicators) were incorporated at each location. Model performance was assessed using 10-fold cross-validation, resulting in a median *R*^2^ of 0.66 for conventional validation.^18^

Each participant’s census tract was identified from the residential address recorded at the time of enrollment using ArcGIS Pro, version 3.0 (Esri), and pollutant concentrations were collected for the enrollment year and the 2 preceding years.^19^ Given the enrollment years for the study were 2010 to 2018, this corresponded to the period of 2008 to 2018. The annual estimates over the 3 years were then averaged to reduce interannual variability, minimizing distortion from atypical local pollution events. Sensitivity analysis considering the 1-year pollutant concentration prior to cancer therapy initiation was also performed.

### Quantitative Echocardiography Outcomes

Quantitative echocardiography was performed at the University of Pennsylvania Center for Quantitative Echocardiography by trained sonographers (A.D., V.E.) blinded to participant characteristics, using the TOMTEC Imaging Systems software (TOMTEC Corporation) according to the American Society of Echocardiography guidelines. This included quantification of cardiac function (LVEF and strain [longitudinal, circumferential]), LV volumes (LV end diastolic volume [LVEDV], LV end systolic volume [LVESV]), LV mass, diastolic function (E/e′ [early diastolic mitral inflow velocity to early diastolic mitral annulus velocity], left atrial [LA] volume), and ventricular-arterial (VA) coupling (Ea/Ees, where *Ea* indicates effective arterial elastance and *Ees*, the slope of the end-systolic pressure-volume relation). VA coupling provides insight into cardiac efficiency. Longitudinal and circumferential strain, both sensitive measures of cardiac systolic function, were reported in absolute values.^20^ The intraobserver coefficient of variation was 4.4% for LVEF, 10.9% for longitudinal strain, and 9.4% for circumferential strain.

In the doxorubicin group, transthoracic echocardiography was performed at baseline, at the completion of paclitaxel (approximately 4 months), annually until year 5, and then biannually. In the doxorubicin plus trastuzumab group, echocardiography was performed at baseline, at the completion of doxorubicin (approximately 2 months), every 3 months during trastuzumab therapy, annually until year 5, and then biannually. In the trastuzumab group, echocardiography was performed at baseline, every 3 months during trastuzumab therapy, annually until year 5, and then biannually (eFigure 1 in Supplement 1).^12^ Cardiac dysfunction was defined as an LVEF decline of 10% or greater from baseline to less than 50%.

### Statistical Analysis

Descriptive statistics were used to characterize participants’ baseline demographic, clinical, and treatment variables overall and by air pollutant tertiles. Normality was assessed using the Shapiro-Wilk test. Nonnormally distributed continuous variables were summarized as medians (IQRs) and normally distributed continuous variables as means (SDs). Differences across air pollutant tertiles were evaluated using the Kruskal-Wallis test for nonnormally distributed variables and 1-way analysis of variance for normally distributed variables. For categorical variables, the Pearson χ^2^ test was used; when expected cell counts were less than 5, the Fisher exact test was applied.

Pollutant levels were evaluated both continuously (per each IQR) and categorically (by tertiles). Covariates that showed a univariable association between air pollution and echocardiographic measures of interest (*P* < .20) or that were hypothesized to be associated with both the exposure and outcome based on clinical judgement^7,19,21,22^ and informed by our group’s prior work^11,12,13,14,15^ were selected as confounders in each model. For the cross-sectional analysis of baseline echocardiographic measures, we used multivariable linear regression, adjusting for covariates including age at baseline, race (included in the analysis as prior research has found associations with echocardiographic outcomes^11^; categories were Black, White, and other [American Indian, Asian, or Pacific Islander and those who selected “other”]), SVI (continuous; a composite measure of structural social determinants of health from the CDC),^17^ hypertension, dyslipidemia, smoking (current or prior vs never), and body mass index (BMI; continuous).

To assess the longitudinal associations between air pollutant measure and echocardiographic indices, generalized estimating equations (GEEs) with an independence correlation structure were used. In our multivariable models, we included each corresponding baseline echocardiographic parameter; age at treatment initiation; race; SVI; treatment regimen (doxorubicin, trastuzumab, or doxorubicin plus trastuzumab); left-sided radiation; baseline status of hypertension, dyslipidemia, smoking, and BMI; an interaction term between treatment regimen and time since cancer therapy initiation (modeled as a cubic spline with 3 *df*); and a robust variance estimator that accounted for within-participant correlation. In these models, we included an interaction term for treatment regimen × time since cancer therapy initiation, given our group’s prior work that suggested the effect of treatment on cardiac function could vary over time.^13^ In an exploratory analysis using the robust Wald test to evaluate for statistical significance, the potential interaction between air pollutant level and cancer therapy regimen, age (≥52 years vs <52 years [mean age of menopausal status in the US]), or cardiovascular risk factors (hypertension, obesity, and dyslipidemia) was explored, given the a priori hypothesis that the potential biologic effects of air pollution on cardiotoxicity could vary according to type of cancer therapy, age, or cardiovascular risk factors. We also explored whether there was an interaction between air pollutant level and time since cancer therapy initiation.

To assess the association between air pollutants and the risk of cardiac dysfunction, defined categorically as an LVEF decline of 10% or more from baseline to less than 50%, cause-specific hazard models with death as a competing risk were used, given the etiologic nature of our research question.^23^ Pollutant exposures were categorized into tertiles, with the lowest tertile as the reference. The same covariate selection criteria as those applied in the cross-sectional and longitudinal analyses were used. The proportional hazards assumption was assessed using Schoenfeld residuals. Univariable and multivariable models were used to estimate hazard ratios (HRs) and 95% CIs. Multivariable models were adjusted for age at baseline; race; treatment regimen; left-sided radiation; baseline status of hypertension, dyslipidemia, smoking, and BMI; and SVI.^17^ Multicollinearity between each air pollutant and SVI was also evaluated. Cumulative incidence curves for cardiac dysfunction across tertiles of air pollutant exposure were plotted after adjustment for covariates, accounting for the competing risk of death. A sensitivity analysis using Fine and Gray’s method to account for the competing risk of death was also performed.^24^ In addition, sensitivity analyses of the cross-sectional and longitudinal associations and a survival analysis were conducted using air pollutant concentrations during the 1-year period prior to enrollment.

To ensure consistency across models, the same covariates were used in each of the air pollutant models. All tests were 2-sided, and *P* < .05 was considered statistically significant. Statistical analyses were conducted with R, version 4.3.2 (R Project for Statistical Computing), from December 1, 2024, to April 30, 2025.

## Results

### Characteristics of the Study Population

Among 580 female participants (median age, 50 years [IQR, 42-58 years]; 142 of 577 [24.6%] Black, 394 of 577 [68.3%] White, and 41 of 577 [7.1%] other race), 268 of 569 (47.1%) had left-sided breast cancer and 311 of 580 (53.6%) had stage II disease (Table 1). Overall, 342 (59.0%) received anthracyclines without trastuzumab, 60 (10.3%) received both anthracyclines and trastuzumab, 178 (30.7%) received trastuzumab without anthracyclines, and 217 (37.4%) underwent left-sided radiotherapy. Among 3642 core-laboratory quantified echocardiograms from the 580 participants, 303 echocardiograms (8.3%) were missing quantified LVEF and 106 (2.9%) were missing relative wall thickness.

**Table 1.** Baseline Characteristics of Patients With Breast Cancer and by PM_2.5_ Exposure

Characteristic	Participants^a^				*P* value
	Total study population (N = 580)	Tertile 1 (least polluted) (n = 193)	Tertile 2 (n = 194)	Tertile 3 (n = 193)	
Age at baseline, y	50 (42-58)	52 (43-61)	50 (41-58)	48 (41-57)	.03
PM_2.5_, median (IQR) [range], μg/m^3^	9.26 (8.49-10.17) [4.08-12.44]	8.06 (7.57-8.49) [4.08-8.80]	9.26 (9.09-9.56) [8.81-9.79]	10.40 (10.17-10.73) [9.80-12.44]	NA
Social Vulnerability Index^b^	0.30 (0.14-0.62)	0.24 (0.12-0.46)	0.28 (0.14-0.57)	0.40 (0.17-0.77)	<.001
Race					
Black	142/577 (24.6)	25/192 (13.0)	41/193 (21.2)	76/192 (39.6)	<.001
White	394/577 (68.3)	154/192 (80.2)	138/193 (71.5)	102/192 (53.1)	
Other^c^	41/577 (7.1)	13/192 (6.8)	14/193 (7.3)	14/192 (7.3)	
Cancer laterality					
Left	268/569 (47.1)	79/188 (42.0)	98/191 (51.3)	91/190 (47.9)	.11
Right	277/569 (48.7)	104/188 (55.3)	86/191 (45.0)	87/190 (45.8)	
Bilateral	24/569 (4.2)	5/188 (2.7)	7/191 (3.7)	12/190 (6.3)	
Cancer stage					
I	127 (21.9)	44 (22.8)	41 (21.1)	42 (21.8)	.74
II	311 (53.6)	103 (53.4)	101 (52.1)	107 (55.4)	
III	133 (22.9)	44 (22.8)	50 (25.8)	39 (20.2)	
IV	9 (1.6)	2 (1.0)	2 (1.0)	5 (2.6)	
Cancer treatment					
Doxorubicin	342 (59.0)	108 (56.0)	115 (59.3)	119 (61.7)	.003
Trastuzumab	178 (30.7)	73 (37.8)	61 (31.4)	44 (22.8)	
Doxorubicin plus trastuzumab	60 (10.3)	12 (6.2)	18 (9.3)	30 (15.5)	
Radiotherapy	409/579 (70.6)	129/193 (66.8)	141/193 (73.1)	139/193 (72.0)	.36
Left-sided radiotherapy	217/579 (37.5)	60/193 (31.1)	80/193 (41.5)	77/193 (39.9)	.08
Cardiovascular history or risk factors					
Current or prior smoking	217/578 (37.5)	71/192 (37.0)	70/193 (36.3)	76/193 (39.4)	.80
Current alcohol intake	318 (54.8)	109 (56.5)	102 (52.6)	107 (55.4)	.73
Sufficient physical activity^d^	70/571 (12.3)	24/190 (12.6)	26/193 (13.5)	20/188 (10.6)	.69
History of hypertension	173 (29.8)	51 (26.4)	53 (27.3)	69 (35.8)	.09
History of diabetes	53/579 (9.2)	16/192 (8.3)	18/194 (9.3)	19/193 (9.8)	.87
History of dyslipidemia	141 (24.3)	60 (31.1)	40 (20.6)	41 (21.2)	.03
Obesity^e^	191 (32.9)	61 (31.6)	60 (30.9)	70 (36.3)	.48
Cardiovascular medications					
ACE inhibitor or ARB use	86 (14.8)	28 (14.5)	26 (13.4)	32 (16.6)	.67
Statin use	81/575 (14.1)	38/189 (20.1)	23/193 (11.9)	20/193 (10.4)	.01
β-Blocker use	54 (9.3)	12 (6.2)	23 (11.9)	19 (9.8)	.15
Clinical measures					
Blood pressure, mm Hg					
Systolic	125 (115-136)	124 (115-136)	126 (116-136)	126 (116-136)	.74
Diastolic	76 (70-82)	76 (71-82)	77 (69-81)	74 (69-82)	.30
Resting heart rate, bpm	78 (70-88)	79 (68-88)	78 (70-85)	80 (72-91)	.02
BMI	26.6 (23.2-31.8)	26.0 (22.7-31.1)	26.2 (23.4-31.7)	28.0 (24.0-32.7)	.04
Echocardiographic measures					
LV systolic function					
LVEF, %	56.4 (52.6-59.8)	58.7 (55.7-62.6)	56.3 (52.8-60.1)	53.7 (51.0-56.7)	<.001
Longitudinal strain, %	17.8 (3.4)	19.5 (3.2)	17.8 (3.3)	16.0 (2.6)	<.001
Circumferential strain, %	26.6 (23.3-30.4)	29.1 (24.2-32.4)	26.5 (23.2-31.2)	25.3 (22.4-28.9)	<.001
LV structure					
LVEDV, indexed, mL/m^2^	55.0 (47.6-63.2)	50.3 (44.4-59.4)	55.4 (47.0-62.1)	59.9 (52.9-66.2)	<.001
LVESV, indexed, mL/m^2^	24.3 (19.7-29.3)	20.9 (16.7-25.1)	24.1 (19.8-29.2)	27.6 (23.3-32.0)	<.001
LV mass, indexed, g/m^2^	65.6 (51.1-76.7)	50.6 (39.9-64.4)	65.6 (52.2-75.6)	72.8 (65.4-81.6)	<.001
Relative wall thickness	0.36 (0.32-0.41)	0.35 (0.31-0.40)	0.36 (0.31-0.41)	0.36 (0.32-0.42)	.19
LV diastolic function					
E/e′	7.2 (5.7-8.8)	7.2 (5.7-8.8)	7.1 (5.7-9.0)	7.1 (5.7-8.5)	.77
LA volume, indexed, mL/m^2^	26.1 (21.0-34.5)	22.4 (18.9-27.5)	24.5 (20.6-29.2)	34.4 (26.6-39.4)	<.001
Diastolic function grade					
Normal	415/543 (76.4)	132/178 (74.2)	146/185 (78.9)	137/180 (76.1)	.34
1	48/543 (8.8)	13/178 (7.3)	15/185 (8.1)	20/180 (11.1)	
≥2	80/543 (14.7)	33/178 (18.5)	24/185 (13.0)	23/180 (12.8)	
Ventricular-arterial coupling					
Ea/Ees	0.90 (0.75-1.07)	0.79 (0.67-0.94)	0.87 (0.78-1.04)	1.01 (0.89-1.20)	<.001

Abbreviations: ACE, angiotensin-converting enzyme; ARB, angiotensin II receptor blocker; BMI, body mass index (calculated as weight in kilograms divided by height in meters squared); bpm, beats per minute; E/e′, early diastolic mitral inflow velocity to early diastolic mitral annulus velocity; Ea/Ees, effective arterial elastance to slope of the end-systolic pressure–volume relation; LA, left atrial; LV, left ventricular; LVEDV, left ventricular end-diastolic volume; LVEF, left ventricular ejection fraction; LVESV, left ventricular end-systolic volume; NA, not applicable; PM_2.5_, fine particulate matter with diameter of 2.5 µm or less.

^a^ Data are presented as mean (SD) for normally distributed continuous variables, median (IQR) for nonnormally distributed continuous variables, and number (percentage) or number out of total number (percentage) for categorical variables.

^b^ Score range of 0 to 1, with higher scores indicating more vulnerability.

^c^ Race was self-reported. Other included participants who identified as American Indian, Asian, or Pacific Islander or those who selected “other.”

^d^ Defined as a Godin moderate-to-vigorous physical activity score of 24 or higher on a scale of 0 to 98.

^e^ Defined as BMI of 30 or higher.

**Figure 1.**
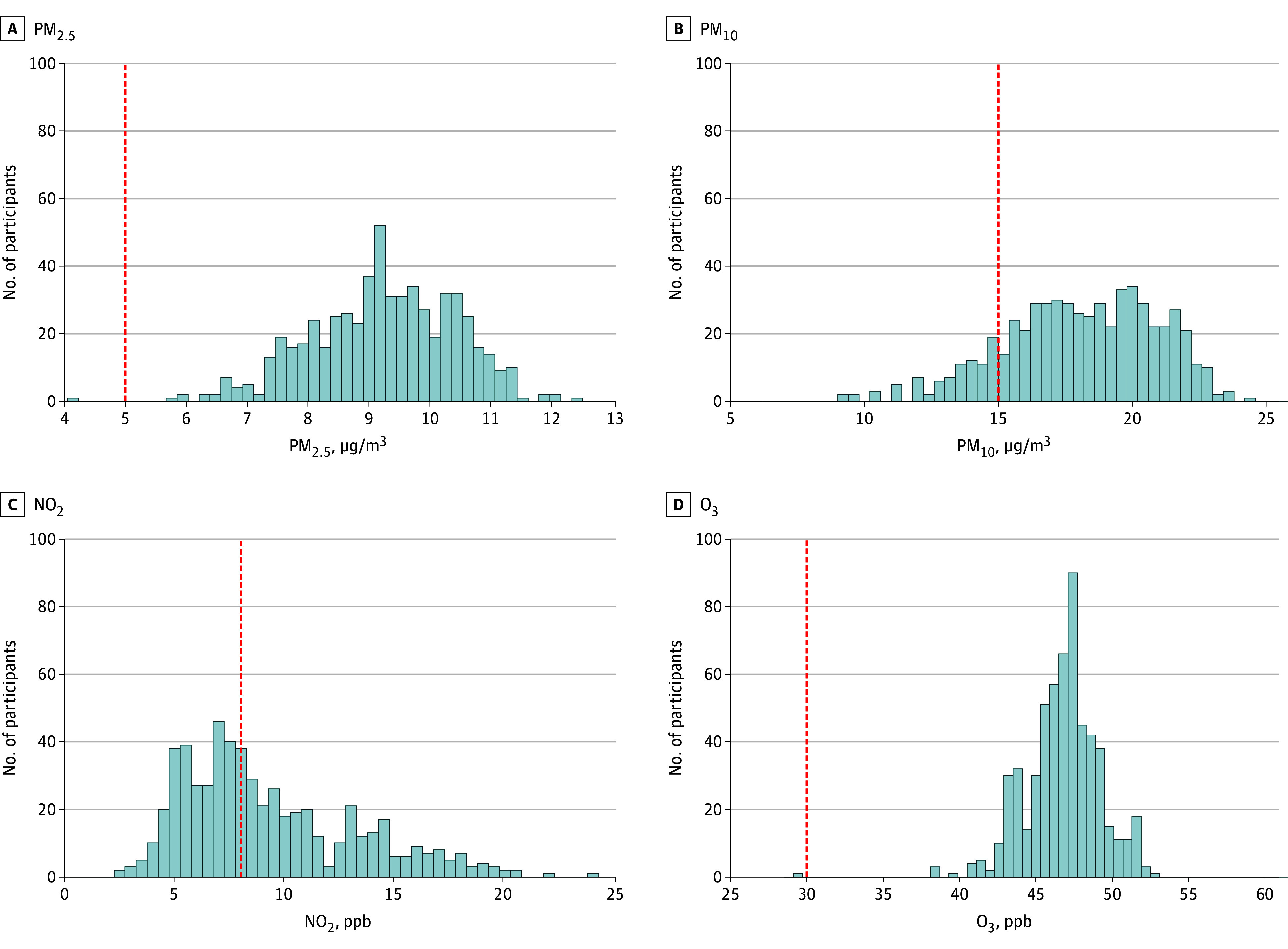
Distribution of Air Pollutants in the Study Population Red dashed lines indicate the World Health Organization air quality guideline threshold for each of the air pollutants: fine particulate matter with diameter of 2.5 µm or less (PM_2.5_) (5 μg/m^3^), particulate matter with diameter of 10 µm or less (PM_10_) (15 μg/m^3^), nitrogen dioxide (NO_2_; 8 parts per billion [ppb], equivalent to 10 μg/m^3^), and ozone (O_3_; 30 ppb, equivalent to 60 μg/m^3^).

There were 445 unique census tract identifiers. At baseline, the median of 3-year mean air pollutant levels among study participants was 9.26 μg/m^3^ (IQR, 8.49-10.17 μg/m^3^; range, 4.08-12.44 μg/m^3^) for PM_2.5_, 18.23 μg/m^3^ (IQR, 16.12-20.23 μg/m^3^; range, 9.12-24.48 μg/m^3^) for PM_10_, 8.20 ppb (IQR, 6.28-11.60 ppb; range, 2.49-23.91 ppb) for NO_2_, and 47.00 ppb (IQR, 45.50-48.19 ppb; range, 29.39-52.96 ppb) for O_3_ (Figure 1). Spearman rank correlation analyses showed that the strongest correlation was between PM_2.5_ and NO_2_ (ρ = 0.72), followed by PM_10_ and NO_2_ (ρ = 0.62) and PM_2.5_ and O_3_ (ρ = 0.52) (eTable 1 in Supplement 1).

Altogether, 579 participants (99.8%) were exposed to PM_2.5_ levels that exceeded the World Health Organization’s guideline of 5 μg/m^3^.^25^ Participants residing in areas with the highest PM_2.5_ concentrations (tertile 3; 9.80-12.44 μg/m^3^) were younger and more likely to be Black, have worse social vulnerability, have higher BMI, and have a greater prevalence of hypertension compared with those in the lowest exposure group (tertile 1; 4.08-8.80 μg/m^3^) (Table 1).

### Cross-Sectional Associations of Air Pollutant Exposure With Echocardiographic Measures

**Figure 2.**
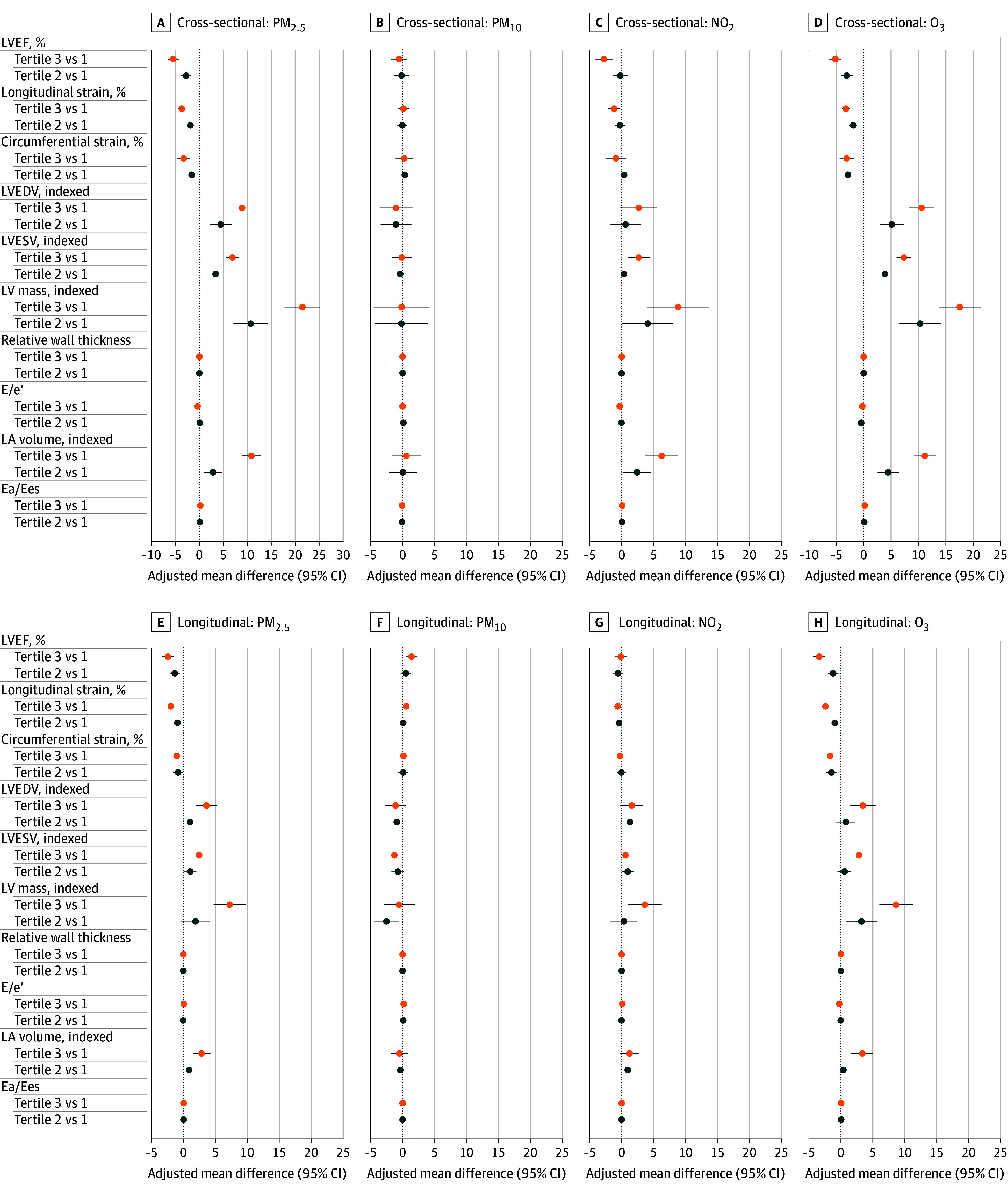
Adjusted Mean Differences in Cardiac Function, Size, and Remodeling by Air Pollutant Exposure at Baseline and After Anthracyclines and/or Trastuzumab Initiation A-D, Baseline. E-H, All time points. Details of analyses are in the Methods. E/e′ indicates early diastolic mitral inflow to mitral annulus velocity; Ea/Ees, effective arterial elastance to slope of the end-systolic pressure–volume relation; LA, left atrial; LV, left ventricular; LVEDV, LV end-diastolic volume; LVEF, LV ejection fraction; LVESV, LV end-systolic volume; NO_2_, nitrogen dioxide; O_3_, ozone; PM_2.5_, fine particulate matter with diameter of 2.5 µm or less; PM_10_, particulate matter with diameter of 10 µm or less.

There were significant baseline (prior to cancer therapy initiation) cross-sectional associations of PM_2.5_, NO_2_, and O_3_ with measures of cardiac function (LVEF, longitudinal strain, and circumferential strain), LV structure (LVEDV, LVESV, and LV mass), LV diastolic function (LA volume), and VA coupling (Ea/Ees) (Figure 2 and eTable 2 in Supplement 1). Each IQR-increment increase in PM_2.5_ concentration (1.68 μg/m^3^) was associated with worse LVEF (−2.9%; 95% CI, −3.6% to −2.3%), longitudinal strain (−2.0%; 95% CI, −2.4% to −1.6%), and circumferential strain (−1.6%; 95% CI, −2.3% to −0.8%); higher LVEDV (5.2 mL/m^2^; 95% CI, 3.8-6.6 mL/m^2^), LVESV (3.9 mL/m^2^; 95% CI, 3.1-4.7 mL/m^2^), and LV mass (11.5 g/m^2^; 95% CI, 9.2-13.7 g/m^2^); and greater VA coupling (Ea/Eas, 0.11; 95% CI, 0.07-0.14) (all *P* < .001). The directionality of these associations was consistent, and all were indicative of worse function and remodeling. Significant associations were also observed for NO_2_ and O_3_ exposure and measures of cardiac structure and function but not for PM_10_. Evaluation of air pollution measures using tertiles found similar results (Figure 2 and eTable 3 in Supplement 1).

### Longitudinal Associations of Air Pollutant Exposure With Cardiac Function, Size, and Remodeling

Over a median follow-up of 3.1 years (IQR, 2.3-3.6 years) that included 3642 core laboratory–quantified echocardiograms, we observed significant longitudinal associations between exposure to PM_2.5_ (median concentration, 9.26 μg/m^3^ [IQR, 8.49-10.17 μg/m^3^]) and O_3_ (median concentration, 47.0 ppb [IQR, 45.50-48.19 ppb]) and multiple measures of cardiac structure and systolic function, strain, and VA coupling (Figure 2 and eTables 4 and 5 in Supplement 1). Each IQR-increment increase in PM_2.5_ concentration (1.68 μg/m^3^) was associated with a change of −1.3% (95% CI, −1.8% to −0.8%) in LVEF, 2.1 mL/m^2^ (95% CI, 1.3-3.0 mL/m^2^) in LVEDV, and 1.4 mL/m^2^ (95% CI, 0.7-2.0 mL/m^2^) in LVESV (all *P* < .001), indicative of worse LV function and remodeling. Similarly, each IQR-increment increase in O_3_ (2.69 ppb) was associated with a change of −1.4% (95% CI, −1.8% to −1.0%; *P* < .001) in LVEF, 1.3 mL/m^2^ (95% CI, 0.4-2.1 mL/m^2^; *P* = .004) in LVEDV, and 1.1 mL/m^2^ (95% CI, 0.5-1.7 mL/m^2^; *P* < .001) in LVESV. Both PM_2.5_ and O_3_ were significantly associated with increases in LV mass (4.8 g/m^2^ [95% CI, 3.1-6.5 g/m^2^] for PM_2.5_ and 3.2 g/m^2^ [95% CI, 2.1-4.3 g/m^2^] for O_3_) and LA volume (2.1 mL/m^2^ [95% CI, 1.3-3.0 mL/m^2^] for PM_2.5_ and 1.5 mL/m^2^ [95% CI, 0.7-2.2 mL/m^2^] for O_3_), with worse longitudinal strain (−1.0% [95% CI, −1.3% to −0.7%] for PM_2.5_ and −1.1% [95% CI, −1.3% to −0.8%] for O_3_), and with worse circumferential strain (−0.6% [95% CI, −1.1% to −0.1%] for PM_2.5_ and −0.8% [95% CI, −1.2% to −0.5%] for O_3_).

Each IQR-increment increase in PM_10_ (4.11 μg/m^3^) was associated with an increase of 0.8% (95% CI, 0.3%-1.3%) in LVEF and a decrease of −0.8 mL/m^2^ (95% CI, −1.5 to −0.1 mL/m^2^) in LVESV. Each IQR-increment increase in NO_2_ (5.31 ppb) was associated with an increase of 2.9 g/m^2^ (95% CI, 1.2-4.6 g/m^2^) in LV mass, a modest worsening in longitudinal strain (−0.3%; 95% CI, −0.6% to −0.1%), and an increase in LA volume (1.4 mL/m^2^; 95% CI, 0.3-2.5 mL/m^2^). However, there were no consistent associations with other parameters of cardiac function or remodeling. Across all air pollutant measures, there was no significant association with the diastolic function measure E/e′.

In an exploratory analysis, the potential interaction of air pollutant exposure and cancer therapy regimen with longitudinal measures of cardiac structure or function was evaluated, and there was no consistent, significant interaction (eTables 6-9 in Supplement 1). Similarly, there was no significant effect modification by age, hypertension, obesity, or dyslipidemia. In an exploratory analysis evaluating the potential interaction between air pollutant level and time since cancer therapy initiation, there was a significant interaction between O_3_ and LVEF, wherein participants with the lowest O_3_ exposure had greater improvements in cardiac function over time (eFigure 2 in Supplement 1).

### Air Pollutant Exposure and Risk of Cardiac Dysfunction

**Figure 3.**
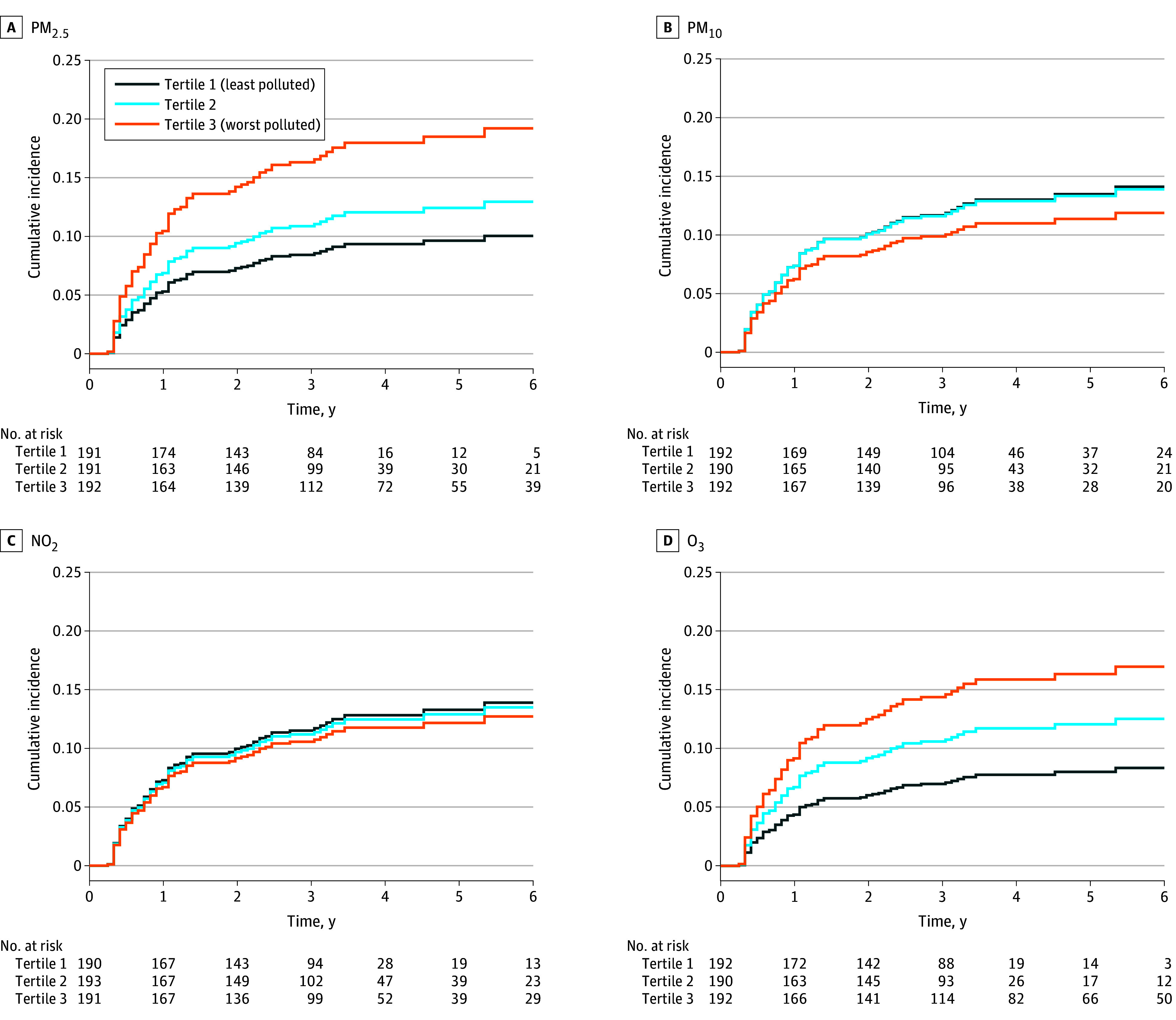
Cardiac Dysfunction According to Air Pollutant Exposure The number of individuals at risk (ie, event-free) at each follow-up time point are presented. Models adjusted for age at baseline, race, treatment regimen, left-sided radiation, baseline hypertension, baseline dyslipidemia, baseline smoking, baseline body mass index, and Social Vulnerability Index. NO_2_ indicates nitrogen dioxide; O_3_, ozone; PM_2.5_, fine particulate matter with diameter of 2.5 µm or less; PM_10_, particulate matter with diameter of 10 µm or less.

Of 574 participants, 98 (17.1%) experienced cardiac dysfunction, defined as a decrease in LVEF of 10% or more to less than 50%. The median time to dysfunction was 0.7 years (IQR, 0.4-1.3 years). Over 1859 person-years of observation, the incidence rate of cardiac dysfunction was 52.7 events per 1000 person-years (Table 2). Moreover, 51 of 574 participants (8.9%) died, with a median time to death of 2.2 years (IQR, 1.5-4.3 years), with 46 of these deaths (90.2%) secondary to cancer (5 [9.8%] had unknown cause). In cause-specific hazard models accounting for death as a competing risk, higher exposure to PM_2.5_ and to O_3_ were each associated with a greater risk of cardiac dysfunction. After adjustment for clinical and sociodemographic factors, participants in the highest tertile of PM_2.5_ (adjusted HR [AHR], 2.03; 95% CI, 1.17-3.52) and those in the highest tertile of O_3_ (AHR, 2.15; 95% CI, 1.23-3.78) had a higher risk of cardiac dysfunction compared with participants in the lowest tertile (Figure 3). Neither PM_10_ (AHR, 0.84; 95% CI, 0.49-1.44) nor NO_2_ (AHR, 0.92; 95% CI, 0.50-1.70) showed significant associations with cardiac dysfunction. Sensitivity analyses using the Fine-Gray method yielded similar results (eTable 10 in Supplement 1).

**Table 2.** Air Pollutant Exposure and Risk of Cardiac Dysfunction With Anthracyclines and/or Trastuzumab Therapy

Pollutant^a^	Participants			PYs	IR per 1000 PYs	Unadjusted HR (95% CI)^c^	Multivariable AHR (95% CI)^d^
	Total, No.	Cardiac dysfunction, No. (%)^b^	Death, No. (%)^b^				
Total study population^e^	574	98 (17.1)	51 (8.9)	1859	52.7	NA	NA
PM_2.5_							
Tertile 1	191	21 (11.0)	11 (5.8)	521	40.3	1 [Reference]	1 [Reference]
Tertile 2	191	28 (14.7)	15 (7.9)	621	45.1	1.31 (0.74-2.31)	1.31 (0.74-2.33)
Tertile 3	192	49 (25.5)	25 (13.0)	717	68.3	2.23 (1.33-3.72)	2.03 (1.17-3.52)
PM_10_							
Tertile 1	192	35 (18.2)	12 (6.2)	653	53.6	1 [Reference]	1 [Reference]
Tertile 2	190	33 (17.4)	24 (12.6)	620	53.3	0.98 (0.61-1.57)	1.00 (0.61-1.64)
Tertile 3	192	30 (15.6)	15 (7.8)	586	51.2	0.91 (0.56-1.48)	0.84 (0.49-1.44)
NO_2_							
Tertile 1	190	31 (16.3)	14 (7.4)	563	55.1	1 [Reference]	1 [Reference]
Tertile 2	193	33 (17.1)	16 (8.3)	650	50.8	1.02 (0.62-1.66)	0.97 (0.59-1.60)
Tertile 3	191	34 (17.8)	21 (11.0)	647	52.6	1.07 (0.66-1.74)	0.92 (0.50-1.70)
O_3_							
Tertile 1	192	20 (10.4)	9 (4.7)	525	38.1	1 [Reference]	1 [Reference]
Tertile 2	190	31 (16.3)	17 (8.9)	557	55.6	1.57 (0.90-2.76)	1.56 (0.88-2.75)
Tertile 3	192	47 (24.5)	25 (13.0)	776	60.5	2.19 (1.29-3.71)	2.15 (1.23-3.78)

Abbreviations: AHR, adjusted hazard ratio; HR, hazard ratio; IR, incidence rate; NA, not applicable; NO_2_, nitrogen dioxide; O_3_, ozone; PM_2.5_, fine particulate matter with diameter of 2.5 µm or less; PM_10_, particulate matter with diameter of 10 µm or less; PY, person-year.

^a^ Tertile 1 indicates least and 3, most polluted.

^b^ Percentages were calculated by multiplying 100 by the number of cardiac dysfunction events (defined by left ventricular ejection fraction changes greater than 10% from baseline to less than 50%) or deaths in each group divided by the total number of study participants in each group.

^c^ A cause-specific hazards model, considering death as a competing risk, was used to calculate unadjusted HRs and 95% CIs.

^d^ A cause-specific hazards model, considering death as a competing risk, was adjusted for age at baseline, race, treatment regimen, left-sided radiation, baseline hypertension, baseline dyslipidemia, baseline smoking status, baseline body mass index, and Social Vulnerability Index.

^e^ Among 580 study participants, 3 (0.5%) were excluded because they did not self-report their race or declined to answer, 1 (0.2%) due to lack of information on left-sided radiation, and 2 (0.3%) due to lack of smoking information. There were 71 patients (12.2%) with a missing quantifiable baseline left ventricular ejection fraction secondary to image quality. In these 71 patients, we imputed the clinical left ventricular ejection fraction value at baseline.

### Sensitivity Analyses of the Exposure Window

Findings from the 1-year air pollution exposure analysis showed cross-sectional and longitudinal associations consistent with those from the primary 3-year model for measures of both cardiac structure and function. Findings were also similar for the risk of cardiac dysfunction.

## Discussion

In this longitudinal prospective cohort study of 580 patients with breast cancer treated with doxorubicin and/or trastuzumab, greater exposure to PM_2.5_ and O_3_ was independently associated with worse LV remodeling and clinically relevant systolic dysfunction. Among the 4 pollutants, PM_2.5_ and O_3_ were the primary exposures associated with worse LVEF and strain and greater LV volumes and mass at baseline (prior to cancer therapy initiation) and over time. These findings provide new knowledge that is specific and relevant to the growing population of patients with breast cancer and offer quantitative insights into the associations between air pollution and measures of cardiac remodeling and function. Although there is a body of literature that supports the association of air pollution with worse clinical outcomes, including HF, the functional basis for these observations—including associations with systolic dysfunction—has not been fully defined.^6,7^ Moreover, this study’s data suggest that fine particulate matter and ozone are the most important ambient air pollution measures to target and modify from a public health perspective.

Our findings suggest that increasing levels of PM_2.5_ are associated with progressive LV remodeling and systolic dysfunction in a population receiving cardiotoxic cancer therapies, which also result in increased oxidative stress, cellular senescence, mitochondrial dysfunction, and injury.^26,27,28^ Mechanistic studies show that PM_2.5_ promotes inflammation, mitophagy, oxidative stress, and accelerated aging and worsens the generation of reactive oxygen species.^7,10,29,30,31^ These effects may also worsen systemic inflammation, which further contributes to adverse cardiac remodeling.^32^ A number of observational studies further support the cardiac effects of air pollution, both in the general population and in individuals with cancer.^19,21,22,33^ For example, in an analysis of Medicare beneficiaries, higher 3-year average PM_2.5_ exposure was associated with a higher relative risk of first hospital admission for CVD including HF,^19^ and investigations in China reported that higher PM_2.5_, NO_2_, and O_3_ levels were associated with a greater prevalence of CVD^21^ and higher risk of cardiovascular mortality.^22^ Similarly, a Canadian cohort study reported 2% to 5% increases in HF risk for incremental increases in PM_2.5_ (per 3.5 μg/m^3^), NO_2_ (per 13.9 μg/m^3^), and O_3_ (per 6.4 μg/m^3^).^33^ However, each of these analyses relied on administrative codes. Our study provides novel insights into the functional, mechanistic changes in cardiac remodeling and the pathophysiology (systolic dysfunction and LV dilation) that precedes clinically overt cardiomyopathy and HF.

Notably, we also found independent associations of higher O_3_ exposure with echocardiographic measures and elevated risk of cardiac dysfunction, paralleling associations with PM_2.5_ exposure. Although O_3_ has been studied predominantly for respiratory and mortality outcomes,^34,35,36^ recent nationwide Chinese cohort studies have reported that long-term O_3_ exposure is associated with a 6% to 25% higher mortality risk from ischemic heart disease per 10-μg/m^3^ incremental increase in O_3_,^37,38^ whereas time-series analyses further showed that short-term spikes in O_3_ levels are also associated with acute hospital admissions for myocardial infarction and HF,^39^ suggesting both acute and cumulative cardiovascular effects. Experimental data indicate that O_3_-induced hypoxic stress, oxidative injury, and systemic inflammation, potentially mediated by pathways such as hypoxia-inducible factor 1, may impair endothelial function and exacerbate arterial stiffness and LV strain.^40,41,42^ Future mechanistic studies are needed to confirm whether these pathways underlie a two-hit model of cardiotoxicity, in which air pollution worsens the adverse remodeling and dysfunction secondary to cancer therapies.

NO_2_ exposure was also associated with worse baseline cardiac measures, including LV function, structural indices, and longitudinal strain. However, NO_2_ was associated with more modest longitudinal changes in cardiac outcomes than were observed for PM_2.5_ and O_3_, and associations with NO_2_ were primarily observed for LV mass, LA volume, and longitudinal strain. As a pollutant commonly produced by fuel combustion from vehicles, power plants, and off-road machinery, NO_2_ is more often implicated in respiratory health outcomes,^43,44^ with more limited evidence relevant to cardiac remodeling in patients with dilated cardiomyopathy.^45^ These mixed findings may reflect source-specific differences in the toxicant mixture that accompanies NO_2_, which may vary according to setting (ie, urban vs rural), yielding heterogenous cardiovascular effects.^46^ PM_10_ showed minimal associations with cardiac outcomes, which is consistent with evidence that smaller particles (PM_2.5_) penetrate more deeply into the respiratory tract with absorption into pulmonary microvasculature.^10^

Our findings provide comprehensive, detailed functional data to support a call to action related to personal, societal, and governmental intervention to improve environmental health.^47,48^ These include strategies to improve air quality in homes and the workplace as well as landscape and transportation reform, including land use and climate change mitigation assessment and use of low-emission vehicles.^49,50^ The Clean Air Act mandates regulatory oversight of pollutants, including the particulate matter and gaseous emissions examined in our study.^51^ Despite notable success in reducing large-scale emissions over the past several decades,^52^ many communities are still exposed to pollutant levels that exceed the World Health Organization’s more recent guidelines of 5 μg/m^3^ for PM_2.5_,^25^ as was the case for 99.8% of the participants in our cohort study (Figure 1). Our findings, therefore, suggest that additional interventions—at the local, state, and federal government level—are of critical necessity to mitigate this modifiable environmental risk, particularly among patients who are at risk for or have chronic diseases such as cancer and CVD. A recent trial suggested that high-efficiency particulate air (HEPA) purifiers are associated with reduced indoor PM_2.5_ exposure and with systolic blood pressure 3 mm Hg lower among participants with brachial systolic blood pressure over 120 mm Hg, with potential implications for cardiovascular health.^53^ This study’s findings provide potential actionable strategies to mitigate the effects of air pollution that can be adapted to the cardio-oncology population.

### Strengths and Limitations

We note a number of strengths of this study, including the sample size, prospective design, comprehensive core laboratory–quantified echocardiographic measurements of cardiac structure and function, and detailed longitudinal follow-up. Moreover, the comprehensive evaluation of the associations between environmental burden and cardiac structure, function, and remodeling, specifically in patients with cancer, provide detailed functional and mechanistic insights into the pathophysiology of disease in this growing population.

Some limitations of this study should also be noted. First, the observational nature of our work precludes determination of causality, and although we provide detailed functional data, our data cannot provide insights into the biologic mechanisms. Second, we used census tract–level pollution estimates, averaging pollutant concentrations over the 3 years preceding and including the baseline. We acknowledge that this might not account for any changes in residence over the 3-year period, although sensitivity analysis from a 1-year exposure period yielded similar findings. Third, although our study took place across multiple centers within our health system, representing a broad range of air pollutant exposures, the patients were still mostly from a single region within the US, and this may have limited the generalizability of our findings. Fourth, we acknowledge that by study design, the number of echocardiograms differed across treatment groups in the first year after cancer therapy initiation, which may result in detection bias. Related to our echocardiography data, there were some measures that were not analyzable secondary to image quality. For key measures, such as LVEF, the proportion of missingness overall was 8.3%, and for measures such as relative wall thickness, missingness was 2.9%. In addition, we did not have a control group of patients without cancer, although prior publications suggest that our effect sizes for cross-sectional associations were larger than those reported in studies of the general population (LV end-diastolic and end-systolic volumes of 0.82 mL to 1.28 mL per 1.32 μg/m^3^ of PM_2.5_, respectively).^54^

## Conclusions

In this cohort study, higher PM_2.5_ and O_3_ exposure was independently associated with adverse LV remodeling and systolic dysfunction in patients with breast cancer receiving anthracyclines and/or trastuzumab-based therapy. These findings provide actionable evidence to support the critical need for the identification and implementation of cardioprotective strategies to mitigate the effects of air pollution on the development of CVD among patients with cancer. A recent study suggestive of the value of HEPA filtration air purifiers provided potential strategies to improve environmental health in this particularly at-risk population.^53^

## Notes

Supplement 1. **eFigure 1.** Study Procedures **eFigure 2.** Air Pollutants and Estimated Longitudinal Changes in Cardiac Function With an Interaction Term Between Air Pollution and Time Since Cancer Therapy Initiation **eTable 1.** Spearman Rank Correlation Tests Across the Air Pollutants **eTable 2.** Cross-Sectional Associations Between Air Pollutant Exposure and Baseline Measures of Cardiac Function, Size, and Remodeling **eTable 3.** Cross-Sectional Association Between Air Pollutant Exposure Tertiles and Baseline Measures of Cardiac Function, Size, and Remodeling **eTable 4.** Longitudinal Associations of Air Pollutant Exposure With Cardiac Function, Size, and Remodeling After Anthracycline and/or Trastuzumab Initiation **eTable 5.** Longitudinal Associations of Air Pollutant Exposure Tertiles With Cardiac Function, Size, and Remodeling After Anthracycline and/or Trastuzumab Initiation **eTable 6.** Longitudinal Associations of PM_2.5_ Exposure Tertiles With Cardiac Function, Size, and Remodeling Stratified by Cancer Treatment Regimen **eTable 7.** Longitudinal Associations of PM_10_ Exposure Tertiles With Cardiac Function, Size, and Remodeling Stratified by Cancer Treatment Regimen **eTable 8.** Longitudinal Associations of NO_2_ Exposure Tertiles With Cardiac Function, Size, and Remodeling Stratified by Cancer Treatment Regimen **eTable 9.** Longitudinal Associations of O_3_ Exposure Tertiles With Cardiac Function, Size, and Remodeling Stratified by Cancer Treatment Regimen **eTable 10.** Sensitivity Analysis—Air Pollutant Exposure and the Risk of Cardiac Dysfunction With Anthracyclines and/or Trastuzumab Therapy (Fine-Gray Model)

Supplement 2.
Data Sharing Statement
