# Supplementary material for: Air Pollution and Cardiac Remodeling and Function in Patients With Breast Cancer
Source: JAMA Netw Open. 2026 Jan 15;9(1):e2552323. doi: 10.1001/jamanetworkopen.2025.52323 (PMC12809362; doi:10.1001/jamanetworkopen.2025.52323)
Supplement: Supplement 1. — eFigure 1. Study Procedures eFigure 2. Air Pollutants and Estimated Longitudinal Changes in Cardiac Function With an Interaction Term Between Air Pollution and Time Since Cancer Therapy Initiation eTable 1. Spearman Rank Correlation Tests Across the Air Pollutants eTable 2. Cross-Sectional Associations Between Air Pollutant Exposure and Baseline Measures of Cardiac Function, Size, and Remodeling eTable 3. Cross-Sectional Association Between Air Pollutant Exposure Tertiles and Baseline Measures of Cardiac Function, Size, and Remodeling eTable 4. Longitudinal Associations of Air Pollutant Exposure With Cardiac Function, Size, and Remodeling After Anthracycline and/or Trastuzumab Initiation eTable 5. Longitudinal Associations of Air Pollutant Exposure Tertiles With Cardiac Function, Size, and Remodeling After Anthracycline and/or Trastuzumab Initiation eTable 6. Longitudinal Associations of PM2.5 Exposure Tertiles With Cardiac Function, Size, and Remodeling Stratified by Cancer Treatment Regimen eTable 7. Longitudinal Associations of PM10 Exposure Tertiles With Cardiac Function, Size, and Remodeling Stratified by Cancer Treatment Regimen eTable 8. Longitudinal Associations of NO2 Exposure Tertiles With Cardiac Function, Size, and Remodeling Stratified by Cancer Treatment Regimen eTable 9. Longitudinal Associations of O3 Exposure Tertiles With Cardiac Function, Size, and Remodeling Stratified by Cancer Treatment Regimen eTable 10. Sensitivity Analysis—Air Pollutant Exposure and the Risk of Cardiac Dysfunction With Anthracyclines and/or Trastuzumab Therapy (Fine-Gray Model) [file jamanetwopen-e2552323-s001.pdf]

Supplementary Online Content

Jung W, Ko K, Smith AM, et al. Air pollution and cardiac remodeling and function in patients with breast cancer. *JAMA Netw Open*. 2026;9(1):e2552323.
doi:10.1001/jamanetworkopen.2025.52323

eFigure 1. Study Procedures

eFigure 2. Air Pollutants and Estimated Longitudinal Changes in Cardiac Function With an Interaction Term Between Air Pollution and Time Since Cancer Therapy Initiation

eTable 1. Spearman Rank Correlation Tests Across the Air Pollutants

eTable 2. Cross-Sectional Associations Between Air Pollutant Exposure and Baseline Measures of Cardiac Function, Size, and Remodeling

eTable 3. Cross-Sectional Association Between Air Pollutant Exposure Tertiles and Baseline Measures of Cardiac Function, Size, and Remodeling

eTable 4. Longitudinal Associations of Air Pollutant Exposure With Cardiac Function, Size, and Remodeling After Anthracycline and/or Trastuzumab Initiation

eTable 5. Longitudinal Associations of Air Pollutant Exposure Tertiles With Cardiac Function, Size, and Remodeling After Anthracycline and/or Trastuzumab Initiation

eTable 6. Longitudinal Associations of $PM_{2.5}$ Exposure Tertiles With Cardiac Function, Size, and Remodeling Stratified by Cancer Treatment Regimen

eTable 7. Longitudinal Associations of PM_{10} Exposure Tertiles With Cardiac Function, Size, and Remodeling Stratified by Cancer Treatment Regimen

eTable 8. Longitudinal Associations of NO_2 Exposure Tertiles With Cardiac Function, Size, and Remodeling Stratified by Cancer Treatment Regimen

eTable 9. Longitudinal Associations of O_3 Exposure Tertiles With Cardiac Function, Size, and Remodeling Stratified by Cancer Treatment Regimen

eTable 10. Sensitivity Analysis—Air Pollutant Exposure and the Risk of Cardiac Dysfunction With Anthracyclines and/or Trastuzumab Therapy (Fine-Gray Model)

This supplementary material has been provided by the authors to give readers additional information about their work.

© 2026 Jung W et al. *JAMA Network Open*.

eFigure 1 Study procedures

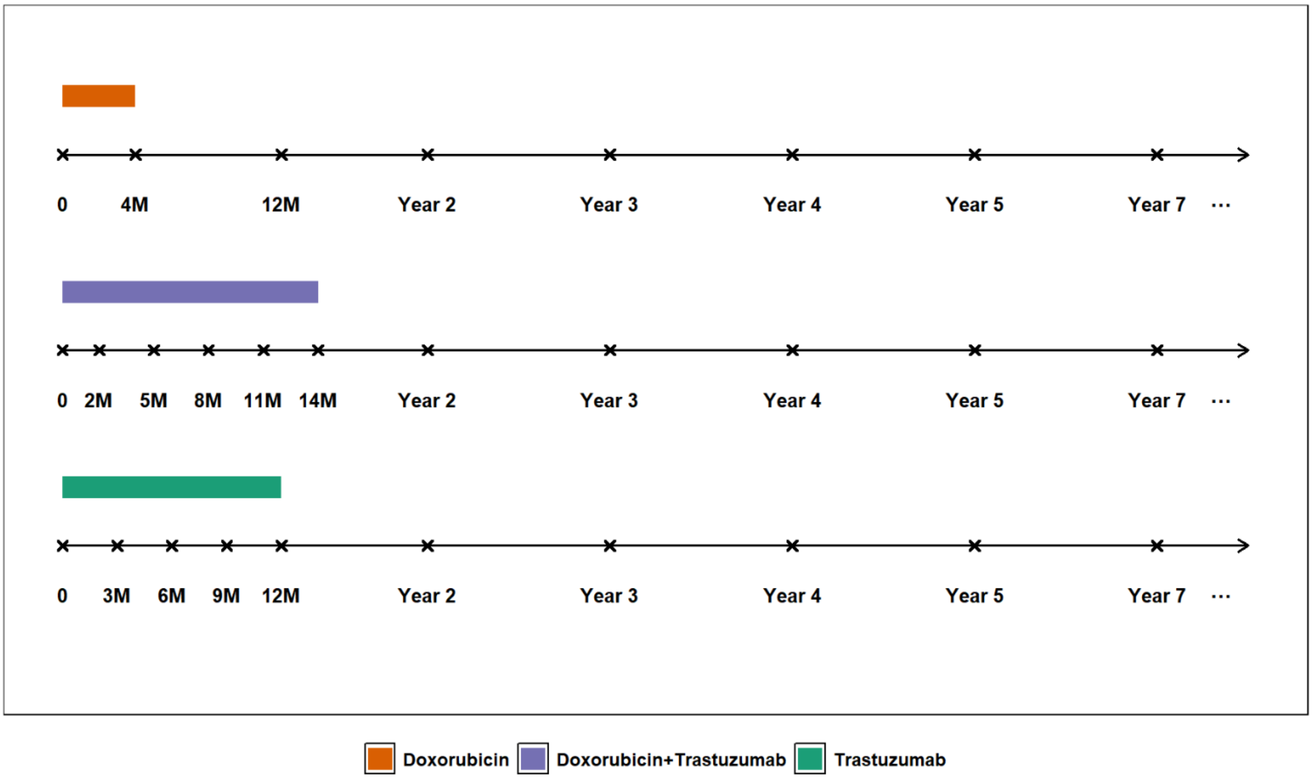

Each colored bar represents the duration of a treatment regimen, and 'X' marks the time points of echocardiography. This analytic cohort includes core lab quantitated echocardiograms obtained over a median (Q1-Q3) follow-up of 3.1 (2.3-3.6) years.

© 2026 Jung W et al. *JAMA Network Open*.

eFigure 2 Air pollutants and estimated longitudinal changes in cardiac function with an interaction term between air pollution and time since cancer therapy initiation

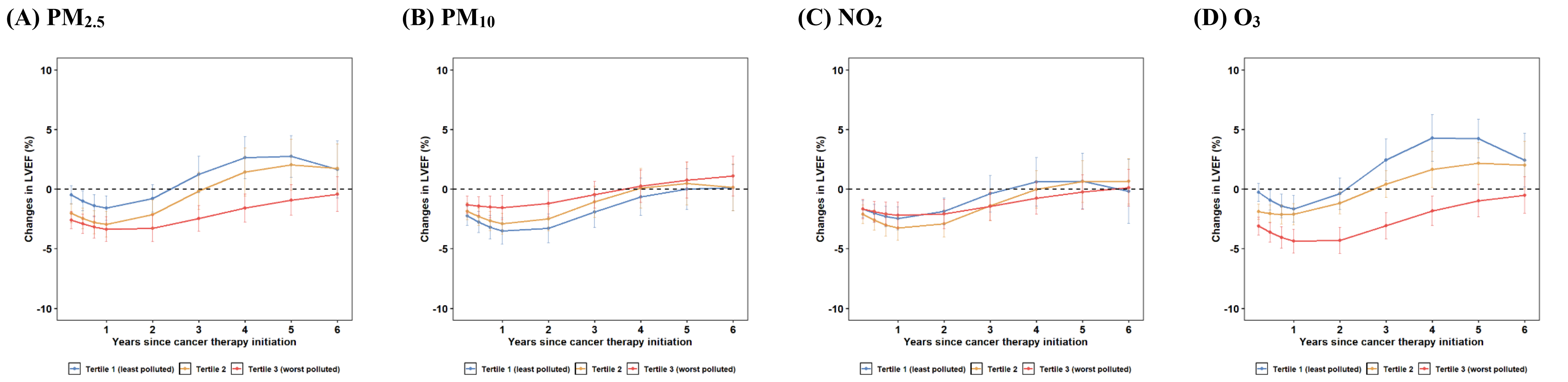

LVEF, left ventricular ejection fraction. Estimated changes in LVEF (%) and 95% confidence intervals at each timepoint (90, 180, 270 days, and each year) are shown. A multivariable linear regression model using generalized estimating equations (GEE) with an independence correlation structure and robust variance estimator was employed to account for repeated measures.

Each model included baseline LVEF (%), air pollutant (tertiles), age at baseline (years), race (Black, White, Other), social vulnerability index (continuous), treatment regimen (Dox, Tras, Dox + Tras), left-sided radiation (no/yes), baseline hypertension (no/yes), baseline dyslipidemia (no/yes), baseline smoking (current or prior, never), baseline body mass index (continuous, kg/m^2), cubic spline of time since cancer therapy initiation with 3 degrees of freedom (df), an interaction term between air pollutant (tertiles)* time since cancer therapy initiation and an interaction term treatment regimen*time since cancer therapy initiation.

© 2026 Jung W et al. *JAMA Network Open*.

eTable 1 Spearman rank correlation tests across the air pollutants

	$PM_{2.5}$	PM_{10}	NO_2	O_3
**$PM_{2.5}$**		0.35	0.72	0.52
**PM_{10}**	0.35		0.62	-0.13
**NO_2**	0.72	0.62		0.03
**O_3**	0.52	-0.13	0.03	

Rho values (ranged from -1 to 1) are presented.

© 2026 Jung W et al. *JAMA Network Open*.

eTable 2 Cross-sectional associations between air pollutant exposure and baseline measures of cardiac function, size, and remodeling

	n	$PM_{2.5}$		PM_{10}		NO_2		O_3	
		Beta-coefficient (95% CI)	P	Beta-coefficient (95% CI)	P	Beta-coefficient (95% CI)	P	Beta-coefficient (95% CI)	P
**LV systolic function**									
LVEF, %	509	-2.9 (-3.6, -2.3)	<0.001	-0.3 (-1.0, 0.5)	0.48	-1.8 (-2.5, -1.0)	<0.001	-2.5 (-3.0, -2.0)	<0.001
Longitudinal strain, %	473	-2.0 (-2.4, -1.6)	<0.001	0.03 (-0.5, 0.5)	0.91	-1.0 (-1.5, -0.5)	<0.001	-1.6 (-2.0, -1.3)	<0.001
Circumferential strain, %	450	-1.6 (-2.3, -0.8)	<0.001	0.2 (-0.6, 1.0)	0.66	-0.7 (-1.5, 0.1)	0.11	-1.6 (-2.2, -1.0)	<0.001
**LV structure**									
LVEDV, indexed, mL/m^2	509	5.2 (3.8, 6.6)	<0.001	-0.3 (-1.8, 1.2)	0.66	2.1 (0.6, 3.6)	0.007	4.7 (3.7, 5.7)	<0.001
LVESV, indexed, mL/m^2	509	3.9 (3.1, 4.7)	<0.001	0.01 (-0.9, 0.9)	0.99	1.9 (1.0, 2.9)	<0.001	3.4 (2.8, 4.0)	<0.001
LV mass, indexed, g/m^2	449	11.5 (9.2, 13.7)	<0.001	-0.5 (-3.0, 2.1)	0.71	5.7 (3.2, 8.2)	<0.001	8.3 (6.6, 10.0)	<0.001
Relative wall thickness	540	0.01 (-0.002, 0.02)	0.14	0.001 (-0.01, 0.01)	0.92	0.001 (-0.001, 0.02)	0.07	0.003 (-0.004, 0.01)	0.36
**LV diastolic function**									
E/e'	527	-0.12 (-0.41, 0.17)	0.42	0.07 (-0.23, 0.37)	0.65	-0.09 (-0.40, 0.21)	0.55	-0.13 (-0.35, 0.09)	0.24
LA volume, indexed, mL/m^2	512	6.3 (5.1, 7.5)	<0.001	0.7 (-0.6, 2.1)	0.30	4.0 (2.6, 5.3)	<0.001	5.1 (4.2, 6.0)	<0.001
**Ventricular-arterial coupling**									
Ea/Ees	418	0.11 (0.07, 0.14)	<0.001	-0.05 (-0.08, -0.01)	0.01	0.04 (-0.003, 0.07)	0.07	0.10 (0.08, 0.13)	<0.001

CI, confidence interval; LVEF, left ventricular ejection fraction; LV, left ventricular; LVEDV, left ventricular end-diastolic volume; LVESV, left ventricular end-systolic volume; E/e', early diastolic mitral inflow velocity to early diastolic mitral annulus velocity; LA, left atrial; Ea/Ees, effective arterial elastance/the slope of the end-systolic pressure-volume relation. n indicated number of echocardiograms.

Beta-coefficients represent the mean difference in each baseline measure in relation to an interquartile range increase in each pollutant (1.68 $\mu g/m^3$ for $PM_{2.5}$, 4.11 $\mu g/m^3$ for PM_{10}, 5.31 ppb for NO_2, 2.69 ppb for O_3).

All results are from a cross-sectional multivariable linear regression adjusting for age at baseline (years), race (Black, White, Other), social vulnerability index (continuous), hypertension (no/yes), dyslipidemia (no/yes), smoking (current or prior, never), and body mass index (continuous, kg/m^2).

© 2026 Jung W et al. *JAMA Network Open*.

eTable 3 Cross-sectional associations between air pollutant exposure tertiles and baseline measures of cardiac function, size, and remodeling

	n	$PM_{2.5}$		PM_{10}		NO_2		O_3	
		**Tertile 2 vs. 1**	**Tertile 3 vs. 1**	**Tertile 2 vs. 1**	**Tertile 3 vs. 1**	**Tertile 2 vs. 1**	**Tertile 3 vs. 1**	**Tertile 2 vs. 1**	**Tertile 3 vs. 1**
**LV systolic function**									
LVEF, %	509	-2.8 (-3.8, -1.7)	-5.4 (-6.5, -4.3)	-0.1 (-1.3, 1.0)	-0.6 (-1.8, 0.7)	-0.2 (-1.4, 0.9)	-2.8 (-4.2, -1.4)	-3.1 (-4.2, -2.0)	-5.2 (-6.3, -4.1)
Longitudinal strain, %	473	-1.9 (-2.6, -1.2)	-3.7 (-4.4, -3.0)	-0.04 (-0.8, 0.7)	0.1 (-0.7, 1.0)	-0.3 (-1.0, 0.5)	-1.2 (-2.1, -0.3)	-1.9 (-2.6, -1.2)	-3.3 (-4.0, -2.5)
Circumferential strain, %	450	-1.6 (-2.9, -0.3)	-3.3 (-4.6, -2.0)	0.3 (-1.0, 1.7)	0.3 (-1.1, 1.6)	0.4 (-0.9, 1.7)	-0.9 (-2.5, 0.6)	-2.9 (-4.1, -1.6)	-3.1 (-4.4, -1.8)
**LV structure**									
LVEDV, indexed, mL/m^2	509	4.5 (2.2, 6.7)	8.9 (6.6, 11.3)	-1.0 (-3.4, 1.4)	-1.0 (-3.6, 1.6)	0.6 (-1.8, 3.0)	2.7 (-0.2, 5.6)	5.1 (2.9, 7.4)	10.6 (8.3, 12.9)
LVESV, indexed, mL/m^2	509	3.4 (2.1, 4.7)	6.9 (5.5, 8.3)	-0.4 (-1.8, 1.1)	-0.1 (-1.7, 1.4)	0.3 (-1.1, 1.8)	2.7 (0.9, 4.4)	3.9 (2.6, 5.2)	7.4 (6.0, 8.7)
LV mass, indexed, g/m^2	449	10.7 (7.1, 14.3)	21.5 (17.8, 25.2)	-0.2 (-4.3, 3.9)	-0.1 (-4.5, 4.2)	4.1 (0.04, 8.1)	8.8 (4.0, 13.6)	10.3 (6.5, 14.1)	17.5 (13.8, 21.3)
Relative wall thickness	540	0.01 (-0.01, 0.02)	0.01 (-0.01, 0.03)	-0.0004 (-0.02, 0.02)	0.0001 (-0.02, 0.02)	-0.0004 (-0.02, 0.02)	0.01 (-0.01, 0.03)	0.003 (-0.01, 0.02)	0.01 (-0.01, 0.02)
**LV diastolic function**									
E/e’	527	0.10 (-0.37, 0.57)	-0.40 (-0.89, 0.09)	0.15 (-0.33, 0.62)	0.02 (-0.49, 0.53)	-0.03 (-0.50, 0.44)	-0.32 (-0.90, 0.26)	-0.45 (-0.92, 0.02)	-0.24 (-0.72, 0.25)
LA volume, indexed, mL/m^2	512	2.9 (0.9, 4.8)	10.8 (8.9, 12.8)	0.1 (-2.1, 2.2)	0.6 (-1.7, 2.9)	2.4 (0.3, 4.5)	6.2 (3.7, 8.8)	4.5 (2.5, 6.4)	11.2 (9.2, 13.2)
**Ventricular-arterial coupling**									
Ea/Ees	418	0.10 (0.05, 0.16)	0.21 (0.15, 0.27)	-0.07 (-0.13, -0.01)	-0.09 (-0.15, -0.03)	0.06 (-0.002, 0.12)	0.07 (-0.004, 0.14)	0.10 (0.04, 0.15)	0.21 (0.15, 0.27)

CI, confidence interval; LVEF, left ventricular ejection fraction; LV, left ventricular; LVEDV, left ventricular end-diastolic volume; LVESV, left ventricular end-systolic volume E/e’, early diastolic mitral inflow velocity to early diastolic mitral annulus velocity; LA, left atrial; Ea/Ees, effective arterial elastance/the slope of the end-systolic pressure-volume relation, n indicated number of echocardiograms.

Beta-coefficients (95% confidence intervals) are shown. Beta-coefficients represent the mean difference for individuals in higher air pollutants tertiles (Tertile 2, 3) compared with those in Tertile 1 (least polluted) in the tertile analysis.

All results are from a cross-sectional multivariable linear regression adjusting for age at baseline (years), race (Black, White, Other), social vulnerability index (continuous), baseline hypertension (no/yes), dyslipidemia (no/yes), smoking (current or prior, never), and body mass index (continuous, kg/m^2).

© 2026 Jung W et al. *JAMA Network Open*.

eTable 4 Longitudinal associations of air pollutant exposure with cardiac function, size, and remodeling after anthracyclines and/or trastuzumab initiation

	n	$PM_{2.5}$		PM_{10}		NO_2		O_3	
		Beta-coefficient (95% CI)	P	Beta-coefficient (95% CI)	P	Beta-coefficient (95% CI)	P	Beta-coefficient (95% CI)	P
**LV systolic function**									
LVEF, %	3,339	-1.3 (-1.8, -0.8)	<0.001	0.8 (0.3, 1.3)	0.004	0.02 (-0.5, 0.5)	0.95	-1.4 (-1.8, -1.0)	<0.001
Longitudinal strain, %	2,901	-1.0 (-1.3, -0.7)	<0.001	0.3 (-0.002, 0.5)	0.05	-0.3 (-0.6, -0.1)	0.02	-1.1 (-1.3, -0.8)	<0.001
Circumferential strain, %	2,712	-0.6 (-1.1, -0.1)	0.01	0.2 (-0.3, 0.6)	0.51	-0.2 (-0.6, 0.2)	0.36	-0.8 (-1.2, -0.5)	<0.001
**LV structure**									
LVEDV, indexed, mL/m^2	3,339	2.1 (1.3, 3.0)	<0.001	-0.7 (-1.7, 0.3)	0.15	1.0 (-0.1, 2.0)	0.071	1.3 (0.4, 2.1)	0.004
LVESV, indexed, mL/m^2	3,339	1.4 (0.7, 2.0)	<0.001	-0.8 (-1.5, -0.1)	0.02	0.2 (-0.4, 0.9)	0.48	1.1 (0.5, 1.7)	<0.001
LV mass, indexed, g/m^2	3,025	4.8 (3.1, 6.5)	<0.001	-0.4 (-1.9, 1.1)	0.62	2.9 (1.2, 4.6)	<0.001	3.2 (2.1, 4.3)	<0.001
Relative wall thickness	3,536	0.001 (-0.003, 0.01)	0.51	0.003 (-0.001, 0.01)	0.13	0.002 (-0.002, 0.01)	0.34	-0.001 (-0.01, 0.002)	0.47
**LV diastolic function**									
E/e'	3,496	0.03 (-0.11, 0.17)	0.70	0.06 (-0.09, 0.21)	0.41	0.07 (-0.07, 0.21)	0.31	-0.07 (-0.19, 0.06)	0.29
LA volume, indexed, mL/m^2	3,330	2.1 (1.3, 3.0)	<0.001	-0.2 (-0.9, 0.6)	0.62	1.4 (0.3, 2.5)	0.02	1.5 (0.7, 2.2)	<0.001
**Ventricular-arterial coupling**									
Ea/Ees	3,059	0.03 (0.01, 0.05)	0.01	-0.01 (-0.03, 0.01)	0.47	0.01 (-0.01, 0.03)	0.58	0.03 (0.01, 0.04)	<0.001

CI, confidence interval; LVEF, left ventricular ejection fraction; LV, left ventricular; LVEDV, left ventricular end-diastolic volume; LVESV, left ventricular end-systolic volume; E/e', early diastolic mitral inflow velocity to early diastolic mitral annulus velocity; LA, left atrial; Ea/Ees, effective arterial elastance/the slope of the end-systolic pressure-volume relation. n indicated number of echocardiograms.

Beta-coefficient represents the average difference in the echocardiographic outcome in relation to an interquartile range increase in each pollutant (1.68 μg/m^3 for $PM_{2.5}$, 4.11 μg/m^3 for PM_{10}, 5.31 ppb for NO_2, 2.69 ppb for O_3) across all observed time points after the initiation of anthracyclines and/or trastuzumab using multivariable linear regression via generalized estimating equations (GEE) with an independence correlation structure and robust variance estimator to account for repeated measures.

Each model included baseline echocardiographic parameters, air pollutant, age at baseline (years), race (Black, White, Other), social vulnerability index (continuous), treatment regimen (Dox, Tras, Dox + Tras), left-sided radiation (no/yes), baseline hypertension (no/yes), baseline dyslipidemia (no/yes), baseline smoking (current or prior, never), baseline body mass index (continuous, kg/m^2), time since cancer therapy initiation modeled as a cubic spline with 3 degrees of freedom (df), and an interaction term between treatment regimen*time since cancer therapy initiation.

© 2026 Jung W et al. *JAMA Network Open*.

eTable 5 Longitudinal associations of air pollutant exposure tertiles with cardiac function, size, and remodeling after anthracycline and/or trastuzumab initiation

	n	$PM_{2.5}$		PM_{10}		NO_2		O_3	
		**Tertile 2 vs. 1**	**Tertile 3 vs. 1**	**Tertile 2 vs. 1**	**Tertile 3 vs. 1**	**Tertile 2 vs. 1**	**Tertile 3 vs. 1**	**Tertile 2 vs. 1**	**Tertile 3 vs. 1**
**LV systolic function**									
LVEF, %	3,339	-1.4 (-2.2, -0.6)	-2.4 (-3.4, -1.5)	0.5 (-0.3, 1.3)	1.4 (0.6, 2.2)	-0.6 (-1.4, 0.2)	-0.1 (-1.1, 0.8)	-1.2 (-2.0, -0.4)	-3.4 (-4.3, -2.5)
Longitudinal strain, %	2,901	-0.9 (-1.4, -0.5)	-2.0 (-2.5, -1.5)	0.1 (-0.3, 0.5)	0.6 (0.1, 1.1)	-0.4 (-0.9, 0.03)	-0.6 (-1.2, -0.06)	-0.9 (-1.4, -0.5)	-2.4 (-2.9, -1.9)
Circumferential strain, %	2,712	-0.8 (-1.6, -0.1)	-1.1 (-1.9, -0.3)	0.1 (-0.7, 0.9)	0.1 (-0.6, 0.8)	-0.04 (-0.8, 0.7)	-0.3 (-1.1, 0.6)	-1.5 (-2.2, -0.7)	-1.7 (-2.4, -0.9)
**LV structure**									
LVEDV, indexed, mL/m^2	3,339	1.0 (-0.4, 2.5)	3.6 (2.0, 5.2)	-0.9 (-2.3, 0.5)	-1.1 (-2.7, 0.5)	1.3 (-0.1, 2.7)	1.6 (-0.2, 3.4)	0.8 (-0.7, 2.3)	3.4 (1.5, 5.4)
LVESV, indexed, mL/m^2	3,339	1.1 (0.1, 2.0)	2.5 (1.3, 3.6)	-0.7 (-1.7, 0.3)	-1.3 (-2.3, -0.2)	0.9 (-0.01, 1.9)	0.6 (-0.6, 1.8)	0.6 (-0.5, 1.6)	2.8 (1.5, 4.2)
LV mass, indexed, g/m^2	3,025	1.9 (-0.3, 4.1)	7.2 (4.7, 9.7)	-2.5 (-4.5, -0.6)	-0.5 (-3.0, 1.9)	0.3 (-1.8, 2.4)	3.6 (1.1, 6.2)	3.2 (0.8, 5.7)	8.6 (6.0, 11.2)
Relative wall thickness	3,536	0.001 (-0.01, 0.01)	0.002 (-0.01, 0.01)	0.001 (-0.01, 0.01)	0.01 (-0.002, 0.01)	0.0002 (-0.01, 0.01)	0.002 (-0.01, 0.01)	-0.004 (-0.01, 0.004)	-0.003 (-0.01, 0.01)
**LV diastolic function**									
E/e’	3,496	-0.06 (-0.31, 0.20)	0.06 (-0.18, 0.30)	0.10 (-0.16, 0.36)	0.18 (-0.08, 0.44)	-0.03 (-0.28, 0.22)	0.10 (-0.17, 0.38)	-0.01 (-0.26, 0.25)	-0.22 (-0.50, 0.05)
LA volume, indexed, mL/m^2	3,330	0.9 (-0.1, 1.9)	2.9 (1.5, 4.2)	-0.3 (-1.4, 0.7)	-0.5 (-1.9, 0.8)	0.9 (-0.1, 2.0)	1.2 (-0.3, 2.7)	0.4 (-0.7, 1.5)	3.3 (1.6, 5.1)
**Ventricular-arterial coupling**									
Ea/Ees	3,059	0.02 (-0.01, 0.05)	0.04 (0.004, 0.08)	0.02 (-0.01, 0.05)	-0.002 (-0.04, 0.03)	-0.01 (-0.04, 0.02)	0.003 (-0.03, 0.04)	0.04 (0.01, 0.08)	0.06 (0.03, 0.10)

CI, confidence interval; LVEF, left ventricular ejection fraction; LV, left ventricular; LVEDV, left ventricular end-diastolic volume; LVESV, left ventricular end-systolic volume; E/e’, early diastolic mitral inflow velocity to early diastolic mitral annulus velocity; LA, left atrial; Ea/Ees, effective arterial elastance/the slope of the end-systolic pressure-volume relation. n indicated number of echocardiograms.

Each beta-coefficient reflects the average difference in the echocardiographic outcome for individuals in higher air pollutant tertiles (Tertile 2, Tertile 3) compared to those in Tertile 1 (least polluted). Both analyses were performed using multivariable linear regression via generalized estimating equations (GEE) with an independence correlation structure and robust variance estimator to account for repeated measures.

Each model included baseline echocardiographic parameters, air pollutant, age at baseline (years), race (Black, White, Other), social vulnerability index (continuous), treatment regimen (Dox, Tras, Dox + Tras), left-sided radiation (no/yes), baseline hypertension (no/yes), baseline smoking (current or prior, never), baseline body mass index (continuous, kg/m^2), baseline dyslipidemia (no/yes), time since cancer therapy initiation modeled as a cubic spline with 3 degrees of freedom (df), and an interaction term between treatment regimen*time since cancer therapy initiation.

© 2026 Jung W et al. *JAMA Network Open*.

eTable 6 Longitudinal associations of $PM_{2.5}$ exposure tertiles with cardiac function, size, and remodeling stratified by cancer treatment regimen

	n	Dox (N = 342)		Tras (N = 178)		Dox + Tras (N = 60)		P*
		Tertile 2 vs. 1	Tertile 3 vs. 1	Tertile 2 vs. 1	Tertile 3 vs. 1	Tertile 2 vs. 1	Tertile 3 vs. 1	
**LV systolic function**								
LVEF, %	3,339	-0.7 (-1.9, 0.5)	-2.4 (-3.7, -1.0)	-1.8 (-3.3, -0.4)	-2.6 (-4.3, -0.9)	-2.9 (-6.4, 0.7)	-2.6 (-5.7, 0.5)	0.39
Longitudinal strain, %	2,901	-0.7 (-1.4, 0.1)	-1.9 (-2.6, -1.1)	-1.0 (-1.7, -0.3)	-2.1 (-2.8, -1.4)	-1.6 (-3.5, 0.3)	-2.2 (-3.9, -0.6)	0.76
Circumferential strain, %	2,712	-0.4 (-1.6, 0.8)	-1.7 (-2.9, -0.5)	-1.1 (-2.5, 0.4)	-0.7 (-2.5, 1.0)	-1.7 (-4.5, 1.0)	0.3 (-2.0, 2.7)	0.007
**LV structure**								
LVEDV, indexed, mL/m^2	3,339	0.9 (-0.9, 2.7)	5.7 (3.8, 7.6)	1.1 (-1.8, 4.1)	1.0 (-2.4, 4.4)	1.8 (-4.9, 8.6)	1.8 (-4.3, 8.0)	0.01
LVESV, indexed, mL/m^2	3,339	0.5 (-0.7, 1.8)	3.3 (1.9, 4.7)	1.4 (-0.4, 3.3)	1.3 (-0.9, 3.6)	2.6 (-2.6, 7.8)	2.1 (-2.5, 6.6)	0.09
LV mass, indexed, g/m^2	3,025	1.5 (-1.5, 4.5)	8.2 (4.6, 11.8)	3.0 (-1.5, 7.6)	8.1 (3.7, 12.5)	-1.1 (-11.0, 8.9)	0.5 (-9.2, 10.3)	0.19
Relative wall thickness	3,536	0.003 (-0.01, 0.01)	0.002 (-0.01, 0.01)	0.002 (-0.01, 0.02)	0.01 (-0.01, 0.02)	-0.01 (-0.03, 0.02)	-0.01 (-0.03, 0.02)	0.83
**LV diastolic function**								
E/e’	3,496	0.05 (-0.3, 0.4)	0.2 (-0.2, 0.6)	-0.2 (-0.8, 0.3)	-0.2 (-0.8, 0.3)	0.2 (-0.5, 1.0)	0.4 (-0.2, 1.0)	0.50
LA volume, indexed, mL/m^2	3,330	1.8 (0.2, 3.5)	3.1 (1.3, 5.0)	0.7 (-1.1, 2.4)	2.0 (-0.6, 4.5)	-2.5 (-6.8, 1.9)	3.4 (-1.7, 8.5)	0.01
**Ventricular-arterial coupling**								
Ea/Ees	3,059	-0.01 (-0.1, 0.04)	0.03 (-0.03, 0.1)	0.1 (-0.01, 0.1)	0.1 (0.02, 0.2)	0.03 (-0.1, 0.2)	-0.002 (-0.2, 0.1)	0.14

LVEF, left ventricular ejection fraction; LV, left ventricular; LVEDV, left ventricular end-diastolic volume; LVESV, left ventricular end-systolic volume; E/e’, early diastolic mitral inflow velocity to early diastolic mitral annulus velocity; LA, left atrial; Ea/Ees, effective arterial elastance/the slope of the end-systolic pressure-volume relation. n indicated number of echocardiograms.

Adjusted mean difference (95% CI) are presented. P^* denotes the P interaction term between cancer treatment regimen and air pollutant; $p < 0.05$ denotes inequality in associations across regimen and a significant interaction. Each adjusted mean difference reflects the average difference in the echocardiographic outcome for individuals in higher $PM_{2.5}$ tertile (Tertile 2, 3) compared to those in Tertile 1 (less polluted). Analysis was performed using multivariable linear regression via generalized estimating equations (GEE) with an independence correlation structure and robust variance estimator to account for repeated measures.

Models included baseline echocardiographic parameters, $PM_{2.5}$, age at baseline (years), race (Black, White, Other), social vulnerability index (continuous), treatment regimen (Dox, Tras, Dox + Tras), left-sided radiation (no/yes), baseline hypertension (no/yes), baseline smoking (current or prior, never), baseline body mass index (continuous, kg/m^2), baseline dyslipidemia (no/yes), time since cancer therapy initiation modeled as a cubic spline with 3 degrees of freedom (df), and an interaction term between treatment regimen*time since cancer therapy initiation.

© 2026 Jung W et al. *JAMA Network Open*.

eTable 7 Longitudinal associations of PM_{10} exposure tertiles with cardiac function, size, and remodeling stratified by cancer treatment regimen

	n	Dox (N = 342)		Tras (N = 178)		Dox + Tras (N = 60)		P*
		Tertile 2 vs. 1	Tertile 3 vs. 1	Tertile 2 vs. 1	Tertile 3 vs. 1	Tertile 2 vs. 1	Tertile 3 vs. 1	
**LV systolic function**								
LVEF, %	3,339	0.03 (-1.2, 1.2)	0.5 (-0.9, 1.8)	0.6 (-1.1, 2.2)	1.4 (-0.1, 3.0)	1.5 (-1.4, 4.4)	4.0 (1.3, 6.7)	0.07
Longitudinal strain, %	2,901	0.4 (-0.3, 1.2)	0.5 (-0.2, 1.3)	-0.2 (-1.1, 0.7)	0.4 (-0.3, 1.2)	-0.5 (-1.6, 0.6)	1.2 (-0.3, 2.7)	0.14
Circumferential strain, %	2,712	-0.04 (-1.1, 1.0)	-0.5 (-1.5, 0.6)	-0.2 (-2.0, 1.6)	0.07 (-1.3, 1.5)	0.8 (-1.4, 3.0)	2.0 (0.03, 4.0)	0.11
**LV structure**								
LVEDV, indexed, mL/m^2	3,339	0.8 (-1.0, 2.6)	1.1 (-1.3, 3.5)	-1.3 (-4.6, 2.0)	-1.9 (-4.9, 1.1)	-5.1 (-10.0, -0.2)	-5.4 (-10.6, -0.2)	0.04
LVESV, indexed, mL/m^2	3,339	0.3 (-0.9, 1.6)	0.2 (-1.2, 1.7)	-0.8 (-3.1, 1.4)	-1.6 (-3.5, 0.4)	-3.4 (-7.0, 0.2)	-4.9 (-8.8, -1.1)	0.04
LV mass, indexed, g/m^2	3,025	-1.6 (-4.2, 1.1)	0.9 (-3.1, 4.9)	-2.2 (-7.1, 2.7)	-0.9 (-5.7, 3.9)	-5.7 (-11.0, -0.3)	-3.7 (-10.0, 2.6)	0.51
Relative wall thickness	3,536	-0.004 (-0.02, 0.01)	-0.001 (-0.01, 0.01)	0.01 (-0.01, 0.02)	0.01 (-0.01, 0.02)	-0.00001 (-0.02, 0.02)	0.02 (-0.002, 0.04)	0.17
**LV diastolic function**								
E/e’	3,496	-0.1 (-0.5, 0.3)	0.02 (-0.4, 0.4)	0.3 (-0.3, 0.9)	0.1 (-0.4, 0.7)	0.3 (-0.3, 1.0)	0.8 (-0.1, 1.6)	0.25
LA volume, indexed, mL/m^2	3,330	0.8 (-0.9, 2.5)	0.3 (-1.9, 2.5)	-1.3 (-3.4, 0.9)	-2.0 (-3.9, 0.001)	-2.0 (-5.9, 1.8)	0.5 (-4.6, 5.5)	0.12
**Ventricular-arterial coupling**								
Ea/Ees	3,059	0.02 (-0.03, 0.1)	0.01 (-0.1, 0.1)	0.04 (-0.02, 0.1)	0.04 (-0.02, 0.1)	-0.03 (-0.1, 0.1)	-0.1 (-0.2, 0.003)	0.10

LVEF, left ventricular ejection fraction; LV, left ventricular; LVEDV, left ventricular end-diastolic volume; LVESV, left ventricular end-systolic volume; E/e’, early diastolic mitral inflow velocity to early diastolic mitral annulus velocity; LA, left atrial; Ea/Ees, effective arterial elastance/the slope of the end-systolic pressure-volume relation. n indicated number of echocardiograms.

Adjusted mean difference (95% CI) are presented. P* denotes the P interaction term between cancer treatment regimen and air pollutant; $p < 0.05$ denotes inequality in associations across regimen and a significant interaction. Each adjusted mean difference reflects the average difference in the echocardiographic outcome for individuals in higher PM_{10} tertile (Tertile 2, 3) compared to those in Tertile 1 (less polluted). Analysis was performed using multivariable linear regression via generalized estimating equations (GEE) with an independence correlation structure and robust variance estimator to account for repeated measures.

Models included baseline echocardiographic parameters, PM_{10}, age at baseline (years), race (Black, White, Other), social vulnerability index (continuous), treatment regimen (Dox, Tras, Dox + Tras), left-sided radiation (no/yes), baseline hypertension (no/yes), baseline smoking (current or prior, never), baseline body mass index (continuous, kg/m^2), baseline dyslipidemia (no/yes), time since cancer therapy initiation modeled as a cubic spline with 3 degrees of freedom (df), and an interaction term between treatment regimen*time since cancer therapy initiation.

© 2026 Jung W et al. *JAMA Network Open*.

eTable 8 Longitudinal associations of NO_2 exposure tertiles with cardiac function, size, and remodeling stratified by cancer treatment regimen

	n	Dox (N = 342)		Tras (N = 178)		Dox + Tras (N = 60)		P*
		Tertile 2 vs. 1	Tertile 3 vs. 1	Tertile 2 vs. 1	Tertile 3 vs. 1	Tertile 2 vs. 1	Tertile 3 vs. 1	
**LV systolic function**								
LVEF, %	3,339	0.4 (-0.9, 1.6)	-0.2 (-1.7, 1.3)	-1.0 (-2.4, 0.4)	-0.3 (-2.1, 1.5)	-2.5 (-5.6, 0.5)	0.4 (-2.3, 3.1)	0.04
Longitudinal strain, %	2,901	-0.3 (-1.1, 0.5)	-0.7 (-1.6, 0.2)	-0.4 (-1.2, 0.5)	-0.5 (-1.4, 0.4)	-1.0 (-2.4, 0.5)	-0.7 (-2.2, 0.9)	0.82
Circumferential strain, %	2,712	0.04 (-1.1, 1.1)	-1.0 (-2.3, 0.3)	0.2 (-1.4, 1.9)	0.3 (-1.3, 1.9)	-0.8 (-3.0, 1.4)	0.9 (-1.3, 3.1)	0.05
**LV structure**								
LVEDV, indexed, mL/m^2	3,339	1.4 (-0.4, 3.2)	3.5 (1.1, 5.9)	0.8 (-2.0, 3.7)	-0.1 (-3.4, 3.2)	1.8 (-4.6, 8.1)	-1.1 (-6.8, 4.5)	0.04
LVESV, indexed, mL/m^2	3,339	0.4 (-0.8, 1.7)	1.5 (-0.2, 3.1)	1.0 (-0.8, 2.8)	0.1 (-2.2, 2.4)	2.1 (-2.5, 6.7)	-1.1 (-5.2, 2.9)	0.04
LV mass, indexed, g/m^2	3,025	1.4 (-1.4, 4.1)	5.5 (1.7, 9.3)	-0.3 (-5.3, 4.7)	1.7 (-3.0, 6.3)	-1.7 (-9.2, 5.9)	1.3 (-6.2, 8.8)	0.48
Relative wall thickness	3,536	0.0004 (-0.01, 0.01)	-0.002 (-0.01, 0.01)	0.01 (-0.01, 0.02)	0.004 (-0.01, 0.02)	-0.01 (-0.04, 0.01)	0.01 (-0.01, 0.03)	0.14
**LV diastolic function**								
E/e'	3,496	-0.1 (-0.5, 0.3)	0.03 (-0.4, 0.4)	0.04 (-0.5, 0.6)	0.1 (-0.5, 0.7)	0.08 (-0.6, 0.8)	0.5 (-0.3, 1.2)	0.77
LA volume, indexed, mL/m^2	3,330	0.5 (-1.2, 2.2)	1.3 (-1.0, 3.7)	1.5 (-0.5, 3.4)	0.3 (-2.0, 2.7)	1.5 (-3.0, 6.0)	2.9 (-2.1, 7.9)	0.52
**Ventricular-arterial coupling**								
Ea/Ees	3,059	-0.03 (-0.1, 0.02)	0.003 (-0.1, 0.1)	0.02 (-0.04, 0.1)	0.03 (-0.04, 0.1)	0.01 (-0.1, 0.1)	-0.1 (-0.2, 0.1)	0.21

LVEF, left ventricular ejection fraction; LV, left ventricular; LVEDV, left ventricular end-diastolic volume; LVESV, left ventricular end-systolic volume; E/e', early diastolic mitral inflow velocity to early diastolic mitral annulus velocity; LA, left atrial; Ea/Ees, effective arterial elastance/the slope of the end-systolic pressure-volume relation. n indicated number of echocardiograms.

Adjusted mean difference (95% CI) are presented. P^* denotes the P interaction term between cancer treatment regimen and air pollutant; $p < 0.05$ denotes inequality in associations across regimen and a significant interaction. Each adjusted mean difference reflects the average difference in the echocardiographic outcome for individuals in higher NO_2 tertile (Tertile 2, 3) compared to those in Tertile 1 (less polluted). Analysis was performed using multivariable linear regression via generalized estimating equations (GEE) with an independence correlation structure and robust variance estimator to account for repeated measures.

Models included baseline echocardiographic parameters, NO_2, age at baseline (years), race (Black, White, Other), social vulnerability index (continuous), treatment regimen (Dox, Tras, Dox + Tras), left-sided radiation (no/yes), baseline hypertension (no/yes), baseline smoking (current or prior, never), baseline body mass index (continuous, kg/m^2), baseline dyslipidemia (no/yes), time since cancer therapy initiation modeled as a cubic spline with 3 degrees of freedom (df), and an interaction term between treatment regimen*time since cancer therapy initiation.

© 2026 Jung W et al. *JAMA Network Open*.

eTable 9 Longitudinal associations of O_3 exposure tertiles with cardiac function, size, and remodeling stratified by cancer treatment regimen

	n	Dox (N = 342)		Tras (N = 178)		Dox + Tras (N = 60)		P*
		Tertile 2 vs. 1	Tertile 3 vs. 1	Tertile 2 vs. 1	Tertile 3 vs. 1	Tertile 2 vs. 1	Tertile 3 vs. 1	
**LV systolic function**								
LVEF, %	3,339	-1.7 (-3.1, -0.3)	-2.7 (-4.2, -1.3)	-1.0 (-2.3, 0.3)	-4.4 (-6.0, -2.9)	0.7 (-2.6, 3.9)	-3.5 (-6.9, -0.2)	0.005
Longitudinal strain, %	2,901	-0.9 (-1.6, -0.2)	-2.3 (-3.0, -1.5)	-1.0 (-1.7, -0.3)	-2.6 (-3.4, -1.9)	-0.8 (-2.9, 1.2)	-2.3 (-4.0, -0.6)	0.91
Circumferential strain, %	2,712	-1.5 (-2.8, -0.2)	-1.3 (-2.5, -0.1)	-2.1 (-3.6, -0.7)	-2.9 (-4.5, -1.2)	1.4 (-1.1, 3.9)	-0.1 (-2.5, 2.2)	0.02
**LV structure**								
LVEDV, indexed, mL/m^2	3,339	1.6 (-1.0, 4.3)	3.6 (0.8, 6.4)	0.9 (-1.6, 3.4)	3.7 (-0.1, 7.4)	-3.9 (-10.7, 3.0)	1.9 (-4.8, 8.5)	0.39
LVESV, indexed, mL/m^2	3,339	1.1 (-0.8, 3.0)	2.5 (0.6, 4.4)	0.6 (-0.9, 2.1)	3.6 (1.2, 6.0)	-2.4 (-7.2, 2.3)	2.4 (-2.5, 7.3)	0.15
LV mass, indexed, g/m^2	3,025	4.2 (-0.1, 8.5)	8.0 (4.1, 11.8)	3.3 (-0.7, 7.2)	12.4 (7.9, 16.8)	-3.4 (-13.4, 6.5)	2.9 (-6.2, 11.9)	0.10
Relative wall thickness	3,536	-0.01 (-0.02, 0.004)	0.002 (-0.01, 0.01)	-0.004 (-0.02, 0.01)	-0.001 (-0.02, 0.02)	0.01 (-0.02, 0.04)	-0.02 (-0.1, 0.01)	0.003
**LV diastolic function**								
E/e’	3,496	-0.1 (-0.5, 0.4)	-0.2 (-0.6, 0.3)	-0.01 (-0.5, 0.5)	-0.5 (-1.1, 0.2)	0.4 (-0.2, 1.0)	0.1 (-0.5, 0.8)	0.42
LA volume, indexed, mL/m^2	3,330	0.7 (-1.0, 2.5)	2.9 (0.3, 5.5)	-0.1 (-1.9, 1.6)	4.3 (1.6, 6.9)	0.6 (-5.5, 6.7)	3.7 (0.4, 7.1)	0.65
**Ventricular-arterial coupling**								
Ea/Ees	3,059	0.02 (-0.03, 0.1)	0.04 (-0.02, 0.1)	0.1 (0.03, 0.1)	0.1 (0.01, 0.1)	-0.02 (-0.2, 0.1)	0.1 (-0.1, 0.2)	0.06

LVEF, left ventricular ejection fraction; LV, left ventricular; LVEDV, left ventricular end-diastolic volume; LVESV, left ventricular end-systolic volume; E/e’, early diastolic mitral inflow velocity to early diastolic mitral annulus velocity; LA, left atrial; Ea/Ees, effective arterial elastance/the slope of the end-systolic pressure-volume relation. n indicated number of echocardiograms.

Adjusted mean difference (95% CI) are presented. P^* denotes the P interaction term between cancer treatment regimen and air pollutant; $p < 0.05$ denotes inequality in associations across regimen and a significant interaction. Each adjusted mean difference reflects the average difference in the echocardiographic outcome for individuals in higher O_3 tertile (Tertile 2, 3) compared to those in Tertile 1 (less polluted). Analysis was performed using multivariable linear regression via generalized estimating equations (GEE) with an independence correlation structure and robust variance estimator to account for repeated measures.

Models included baseline echocardiographic parameters, O_3, age at baseline (years), race (Black, White, Other), social vulnerability index (continuous), treatment regimen (Dox, Tras, Dox + Tras), left-sided radiation (no/yes), baseline hypertension (no/yes), baseline smoking (current or prior, never), baseline body mass index (continuous, kg/m^2), baseline dyslipidemia (no/yes), time since cancer therapy initiation modeled as a cubic spline with 3 degrees of freedom (df), and an interaction term between treatment regimen*time since cancer therapy initiation.

© 2026 Jung W et al. *JAMA Network Open*.

eTable 10 Sensitivity analysis - Air pollutant exposure and the risk of cardiac dysfunction with anthracyclines and/or trastuzumab therapy (Fine-Gray Model)

		Participants	Cardiac dysfunction (%) [b]	Death (%) [b]	PYs	IR (per 10^3 PYs)	Unadjusted [c] sdHR (95% CI)	Multivariable adjusted [d] sdHR (95% CI)
**Total study population [a]**		574	98 (17.1)	51 (8.9)	1,859	52.7	NA	NA
**$PM_{2.5}$**	Tertile 1 (least polluted)	191	21 (11.0)	11 (5.8)	521	40.3	1 (Reference)	1 (Reference)
	Tertile 2	191	28 (14.7)	15 (7.9)	621	45.1	1.31 (0.74, 2.32)	1.30 (0.72, 2.36)
	Tertile 3 (worst polluted)	192	49 (25.5)	25 (13.0)	717	68.3	2.23 (1.34, 3.69)	2.04 (1.17, 3.55)
**PM_{10}**	Tertile 1 (least polluted)	192	35 (18.2)	12 (6.2)	653	53.6	1 (Reference)	1 (Reference)
	Tertile 2	190	33 (17.4)	24 (12.6)	620	53.3	0.96 (0.60, 1.54)	0.99 (0.61, 1.61)
	Tertile 3 (worst polluted)	192	30 (15.6)	15 (7.8)	586	51.2	0.90 (0.55, 1.47)	0.83 (0.49, 1.42)
**NO_2**	Tertile 1 (least polluted)	190	31 (16.3)	14 (7.4)	563	55.1	1 (Reference)	1 (Reference)
	Tertile 2	193	33 (17.1)	16 (8.3)	650	50.8	1.02 (0.62, 1.67)	0.98 (0.60, 1.60)
	Tertile 3 (worst polluted)	191	34 (17.8)	21 (11.0)	647	52.6	1.07 (0.65, 1.73)	0.93 (0.49, 1.74)
**O_3**	Tertile 1 (least polluted)	192	20 (10.4)	9 (4.7)	525	38.1	1 (Reference)	1 (Reference)
	Tertile 2	190	31 (16.3)	17 (8.9)	557	55.6	1.55 (0.88, 2.73)	1.54 (0.86, 2.75)
	Tertile 3 (worst polluted)	192	47 (24.5)	25 (13.0)	776	60.5	2.19 (1.30, 3.70)	2.18 (1.24, 3.82)

PY, person-years; IR, incidence rate; sdHR, sub-distribution hazard ratio; CI, confidence interval; NA, not applicable.

[a] Among 580 study participants, 3 patients were excluded because they did not self-report their race or declined to answer, 1 patient was excluded due to lack of information on left-sided radiation, and 2 patients were excluded due to lack of smoking information. There were 71 patients with a missing quantitated baseline LVEF secondary to image quality. In these 71 patients, we imputed the clinical LVEF value at baseline.

[b] Percentage calculated by multiplying 100 by number of cardiac dysfunction events (cardiac dysfunction defined by left ventricular ejection fraction changes greater than 10% from baseline to less than 50%) or deaths in each group divided by total number of study participants in each group

[c] A Fine-Gray model, considering death as a competing risk, was used to calculate sub-distribution hazard ratios and confidence intervals.

[d] A Fine-Gray model, considering death as a competing risk, adjusted for age at baseline (years), race (Black, White, Other), treatment regimen (Dox, Tras, Dox + Tras), left-sided radiation (no/yes), baseline hypertension (no/yes), baseline smoking (current or prior, never), baseline body mass index (continuous, kg/m^2), baseline dyslipidemia (no/yes), and social vulnerability index (continuous).

© 2026 Jung W et al. *JAMA Network Open*.
