# Supplementary material for: Air Pollution and Cardiac Remodeling and Function in Patients With Breast Cancer
Source: JAMA Netw Open. 2026 Jan 15;9(1):e2552323. doi: 10.1001/jamanetworkopen.2025.52323 (PMC12809362; doi:10.1001/jamanetworkopen.2025.52323)
Supplement: Supplement 2. — Data Sharing Statement [file jamanetwopen-e2552323-s002.pdf]

Data Sharing Statement

Jung. Air Pollution and Cardiac Remodeling and Function in Patients With Breast Cancer. *JAMA Netw Open*. Published January 15, 2026. doi:10.1001/jamanetworkopen.2025.52323

Data
Data available: Yes
Data types: Deidentified participant data, Other (please specify)
Additional Information: The datasets used and/or analyzed during the current study are available from the corresponding author on reasonable request.
How to access data: The datasets used and/or analyzed during the current study are available from the corresponding author on reasonable request. (bonnie.ky@pennmedicine.upenn.edu)
When available: With publication

Supporting Documents
Document types: Informed consent form
How to access documents: The datasets used and/or analyzed during the current study are available from the corresponding author on reasonable request. (bonnie.ky@pennmedicine.upenn.edu)
When available: With publication

Additional Information
Who can access the data: researchers whose proposed use of the data has been approved
Types of analyses: for a specified purpose
Mechanisms of data availability: The datasets used and/or analyzed during the current study are available from the corresponding author on reasonable request. (bonnie.ky@pennmedicine.upenn.edu)
